# Computational Super-Resolution: An Odyssey in Harnessing Priors to Enhance Optical Microscopy Resolution

**DOI:** 10.1021/acs.analchem.4c07047

**Published:** 2025-02-27

**Authors:** Wenfeng Tian, Riwang Chen, Liangyi Chen

**Affiliations:** † New Cornerstone Science Laboratory, National Biomedical Imaging Center, State Key Laboratory of Membrane Biology, Institute of Molecular Medicine, College of Future Technology, Peking University, Beijing 100871, China; ‡ New Cornerstone Science Laboratory, State Key Laboratory of Membrane Biology, Academy for Advanced Interdisciplinary Studies, Peking University, Beijing 100871, China; ∥ New Cornerstone Science Laboratory, National Biomedical Imaging Center, State Key Laboratory of Membrane Biology, Beijing Key Laboratory of Cardiometabolic Molecular Medicine, Institute of Molecular Medicine, College of Future Technology, Center for Life Sciences, Peking University, Beijing 100871, China; ∇ PKU-IDG/McGovern Institute for Brain Research, Beijing 100084, China

“Seeing is believing.” Fluorescence microscopy facilitates highly specific imaging of bioprocesses with detailed spatiotemporal information. However, classic optical microscopy is fundamentally constrained by the diffraction limit, preventing fine details in organelles and macromolecules from being resolved.^1^ This limitation has led to the development of super-resolution (SR) methods.

The most well-known SR methods include super-resolution fluorescence microscopy^2^ such as stimulated emission depletion microscopy (STED)^3^ and single-molecule localization microscopy (SMLM),^4,5^ which were awarded with the Nobel Prize in Chemistry in 2014. Although not well recognized in 2014, structured illumination microscopy (SIM)^6^ is also a prominent SR technique for live-cell SR imaging. Moreover, other methods such as super-resolution optical fluctuation imaging (SOFI)^7^ and expansion microscopy^8^ have also emerged recently.

A holistic view of dynamic biological phenomena in their native states is essential for understanding life, necessitating live-cell SR imaging capabilities. However, the live-cell SR imaging scheme remains constrained due to the compromise between the imaging speed and the resolution. As many SR imaging methods rely on prolonged exposure to collect more photons to improve resolution, an increase in physical resolution may lead to blurred images of fast-moving subcellular structures in live-cell imaging conditions.^9^ Meanwhile, fluorescent molecules emit a finite number of photons before irreversibly photobleaching, and biological samples can withstand only limited light exposure. In this context, the photon budget—the maximum flux of photons that can be emitted and detected—limits the practical resolution achievable in live cells. Enhancing resolution often requires excessive illumination power, causing compromised sample integrity. Thus, live-cell SR imaging entails a delicate balance among spatial resolution, the signal-to-noise ratio (SNR), and structural integrity.

Recently, *computational super-resolution* (CSR), which enhances image resolution through computational processes without modifying microscope hardware, has expanded the possibilities for capturing detailed biological information in live systems. CSR enables SR in a postprocessing phase, preserving imaging speed and sample integrity while extending the potential imaging properties set by the photon budget. Advances in conventional analytical algorithms^10^ and deep learning models^11^ have significantly broadened CSR’s capabilities, enabling applications across diverse imaging modalities.^12−14^

Historically, the idea of CSR predates the advent of SR microscopy,^15^ once referred to as “data inversion” or “mathematical band exploration”.^16^ Despite its potential, CSR remains under-appreciated within the biological research community. One primary reason is the absence of a unified concept, resulting in fragmented terminology such as deconvolution algorithms, image deblurring, noise filtering, and artificial intelligence methods. After data inversion was summarized early,^16^ traditional deconvolution methods for fluorescence imaging were discussed in 2006.^17^ Regarding the mathematical concepts of optical super-resolution, Lindberg put forth a review in 2012,^18^ and the latest one focusing on algorithms appeared in 2020.^19^ Although these reviews are insightful, they focus on only specific aspects and do not incorporate the latest advances. To the best of the authors’ knowledge, there has been no comprehensive review that explicitly defines CSR in microscopy or integrates existing methods under a single framework, as listed here in Figure 1.

**Figure 1:**
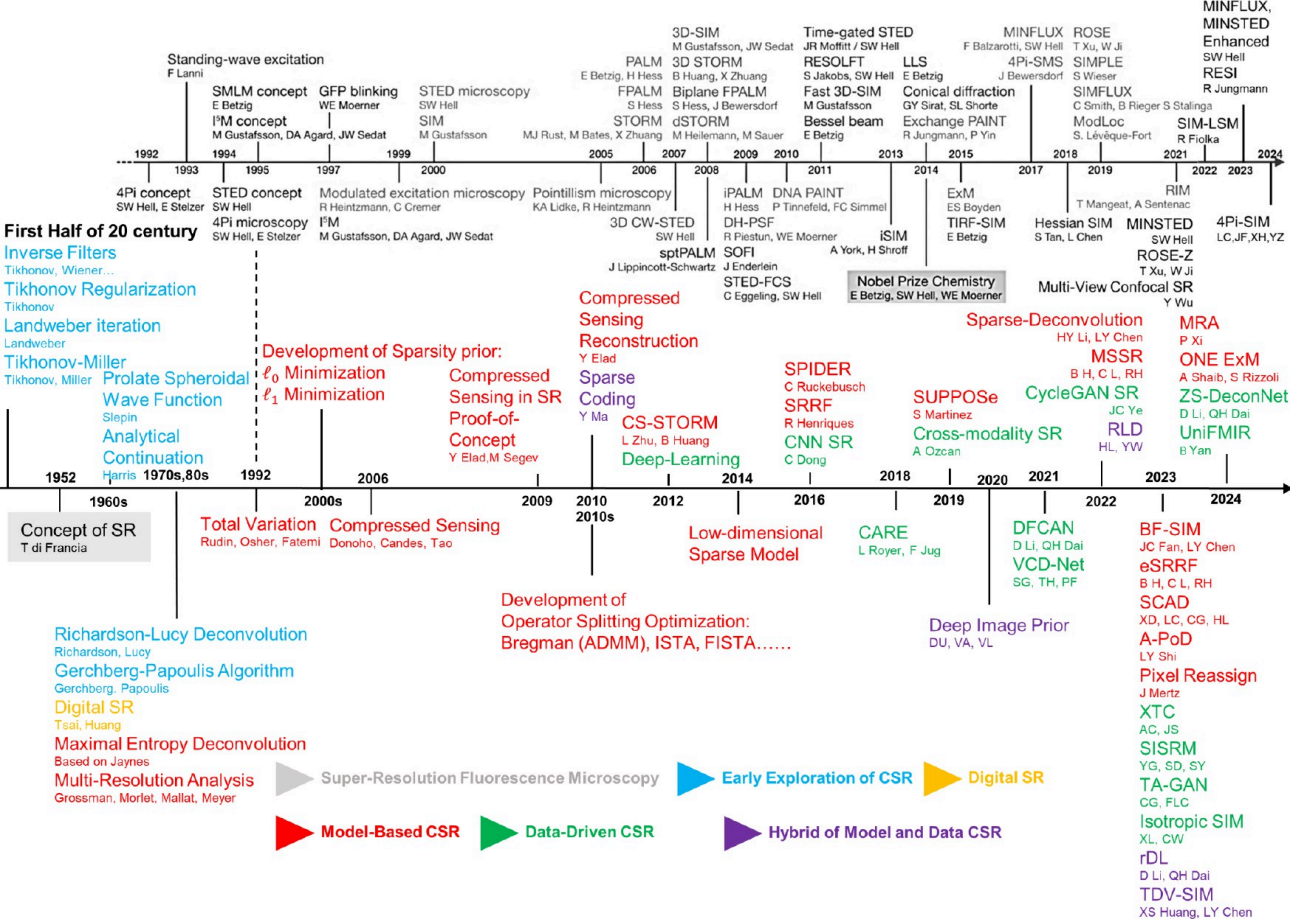
Key milestones in the development of super-resolution fluorescence microscopy and CSR methods. All of the CSR methods in the diagram are covered in this Review. Computational techniques dedicated to a specific SR fluorescence microscopy are not listed. Adapted in part with permission from ref (25). Copyright Prakash Kirti, Diederich Benedict, Heintzmann Rainer, and Schermelleh Lothar, licensed under a Creative Common Attribution (CC BY) 4.0 license.

Another barrier lies in the interdisciplinary nature of CSR, existing at the intersection of computation, mathematics, and physics. Mastering CSR requires an understanding of imaging systems,^20^ optimization,^21^ and deep learning,^22^ making it akin to a “black box” for many microscopists and biologists or, worse, often misinterpreted as mere image enhancement or “photoshopping”.

This Review aims to address these challenges by conceptualizing CSR as a framework that exploits priors to recover high-frequency spectrum information and organize its existing methods into a unified system. We will provide an in-depth explanation of the principles of CSR, with particular emphasis on the historical evolution of CSR methods. Equally important, we highlight the biological applications of CSR, either alone or in combination with other SR techniques, with a focus on overcoming the limitations imposed by photon budgets. We hope this Review serves as a Hitchhiker’s Guide to CSR for microscopists and biologists alike.

The Review is structured as follows: The first section defines resolution and SR, establishing a precise definition of the CSR and introducing its core concepts. This foundational section sets the stage for the remainder of the Review. Following this, we explore CSR methods from a historical perspective, dividing the development into four overlapping stages: early explorations, model-based CSR, data-driven CSR, and the latest advancements. The latter two sections discuss applications of CSR in live-cell SR imaging, biological discovery, and objective resolution evaluation in CSR. We conclude with a discussion of the challenges and future prospects of CSR. Table 1 summarizes all of the discussed methods along with their pros and cons.

**Table 1:** CSR Methods in This Review

methods	priors	algorithms	advantages	drawbacks	application
inverse filters	imaging is a linear system	linear system	easy-to-use	noise infeasible	deblurring, denoising
analytical continuation	finite spatial size	not restricted	theoretical guarantees	noise infeasible	low practicality
PWSFs decompose	finite spatial size	truncated PWSFs	improvement of noise robust	still noise infeasible	low practicality
Gerchberg–Papoulis Algorithm	finite spatial size	iterative algorithm	theoretical guarantees, improvement of noise robust	still noise infeasible	SR, phase retrival^155^
Landweber iteration	smoothness	iterative algorithm	improvement of noise robust	still noise overfitting, iteration number choose	mainly noise filter
Tikhonov–Miller	smoothness	iterative algorithm	improvement of noise robust	still noise overfitting, iteration number choose	noise filter, deconvolution, background light remove
Richardson–Lucy Deconvolution	poisson noise model	iterative algorithm	positive, photon conserving	noise susceptibility; pattern artifacts	general, mainly deconvolution, deblurring, denoising
maximal entropy deconvolution	maximal entropy of data information	iterative algorithm	positive, smooth	resolution impaired	deconvolution
total variation	edges of image are sparse	proximal operator splitting algorithm	edge preserving; efficient algorithms	cartoon-like artifacts	mainly denoising
multi-resolution analysis	multiresolution nature in images	not specifically	theoretical guarantees	nonstationarity sensitivity, edge effects	general, mainly denoising
MRA^75^	edge continuity and across-edge sparsity	proximal operator splitting algorithm	fidelity	rely on the prior	SR, deconvolution
*l*_0_ minimization	sparsity prior	specific algorithms	theoretical guarantees	no efficient algorithm	signal processing, including SR
*l*_1_ minimization		ISTA or FISTA(see Supporting Information)	theoretical guarantees; efficient algorithms	not general feasible in SR	signal processing, including SR
analytical framework (see Supporting Information)		proximal operator splitting algorithm	generality	handcraft prior	general, most low-level processing
sparse coding (see Supporting Information)		proximal operator splitting algorithm; machine learning	generality	handcraft prior	general, most low-level processing
sparse-deconvolution^10^	continuation and sparsity	proximal operator splitting algorithm	versatility in SR	parameters tuning, computation burden	SR, background light remove
Wang et al.^11^	data prior	supervised learning	high performance, fast and flexible for deployment	data dependency, generalization, stability, black-box	cross-modalities SR
RCAN^121^					denoising, SR
DFCAN^110^					SR
XTC^114^					SR
VCD-Net^115^					light-field microscopy reconstruction and isotropic resolution
Task-GAN^125^					SR
SISRM^111^					SR
rDL^139^					SIM SR
UniFMIR^146^					foundation model
RLD^156^					improve RL, parameters autotuning, acceleration
ZS-DeconvNet^112^		unsupervised learning	partially alleviate data collection issues	lack one-size-fits-all approach	SR
Li et al.^113^					isotropic resolution
Park et al.^127^					
Deep image prior	neural network architecture	classic optimization	totally alleviate data collection issues	early stopping issue, prone to low frequency	general
TDV-SIM^154^	data prior + SIM	SIM reconstruction and ANN optimization	suppressing artifacts	computation burden	SIM SR
SUPPOSe^157^ and A-PoD^13^	virtual points	genetic algorithms and Adam	clever prior model	nonconvex, points number determination	SR, especially in Raman microscopy
SRRF and eSRRF	fluorescence fluctuation	specific operation	versatility	need fluctuation	SOFI and other multi-images SR
MSSR^158^	process of photon emission	derived from the mean shift (MS) theory	versatility in SR	artifacts	SR
Zhao et al.^159^	High resolution means narrower PSF	pixel-reassignment	easy-to-use	lack fidelity	SR

This Review strives to balance rigorous principles with accessibility for application-focused readers. Given the interdisciplinary nature of CSR, the use of mathematical language is essential. Yet, we have minimized technical details in the main text and provided critical mathematical content in the Supporting Information. Readers only need a basic understanding of linear algebra^23^ and Fourier transform analysis^24^ to follow the key ideas.

Lastly, two points regarding the scope of this Review need emphasis:

First, this Review focuses specifically on CSR. We do not discuss more general microscopy image restoration methods, which are covered in earlier^26^ and recent reviews.^27^ Similarly, we exclude computational techniques closely associated with certain SR fluorescence microscopy such as localization, clustering, and drift estimation. Readers interested in these computational techniques may refer to refs (28) and (29).

Second, SR is a frequently misunderstood term, as it encompasses various definitions. Notably, SR in computer vision,^30−33^ referred to as natural image super-resolution, is distinct from CSR in microscopy. For the sake of completeness, the Supporting Information elaborates on the distinction between CSR and natural image SR.

We invite readers to explore the exciting historical developments of the CSR through this lens.

## Notions

In this Review, we use the terms “image” and “signal” interchangeably. Lowercase italic letters *x* denote continuous function (or random variable), lowercase Roman letters x denote discrete (digital) image. Lowercase bold Roman **x** represents a vector and uppercase bold **X** represents a matrix. Because discrete image can be mathematically represented by a vector, x and bold x would be used intercahngeably. A dash on the letter x̅ denotes its Fourier transform, a star on the letter x* denotes the result of a certain algorithm; the inner product of two functions *f(r)* and *g(r)* is ⟨*f, g*⟩≔ ∫ *f*(*r*)*g*(*r*) d*r*. The  norm of n-dimension vector **x** is . The total number of nonzero elements in vector **x** corresponds to its *l*_0_ norm, although it does not meet the mathematical definition of a norm. For matrix **A**, its null space is null (**A**):{**x**|**A**·**x** = 0 }; the Fourier matrix **F** of order n is a *n* × *n* matrix in which **F**_*jk*_ = *e*^2π*ijk*/*n*^ where *j, k* ∈ {0,1,···, *n* – 1} and *i*^2^ = −1; **I** denotes the identity matrix; **U**^T^ is the transpose of **U**; **U**^†^ is the conjugate transpose of **U**; and matrix **U** is unitary if **U**^†^**U** = **UU**^†^ = **I**.

## Concepts and Principles of CSR

This section serves as the foundational basis for the Review. It begins by defining the concepts of resolution and SR and then formalizes the notion of CSR. Next, it analyzes the core challenges of CSR and explains underlying principles, with a focus on the use of priors. Finally, it summarizes the main approaches for modeling priors.

### Imaging and Resolution

This Review uses fluorescence microscopy as an example, although CSR is applicable to other imaging modalities. Fluorescence microscopy relies on a laser to excite fluorophores, which emit fluorescence signals collected by a photomultiplier or a camera to form an image. Despite its mechanical complexity, the imaging process of a fluorescence microscope can be abstracted as a 4f optical system, explained below.

**Figure 2:**
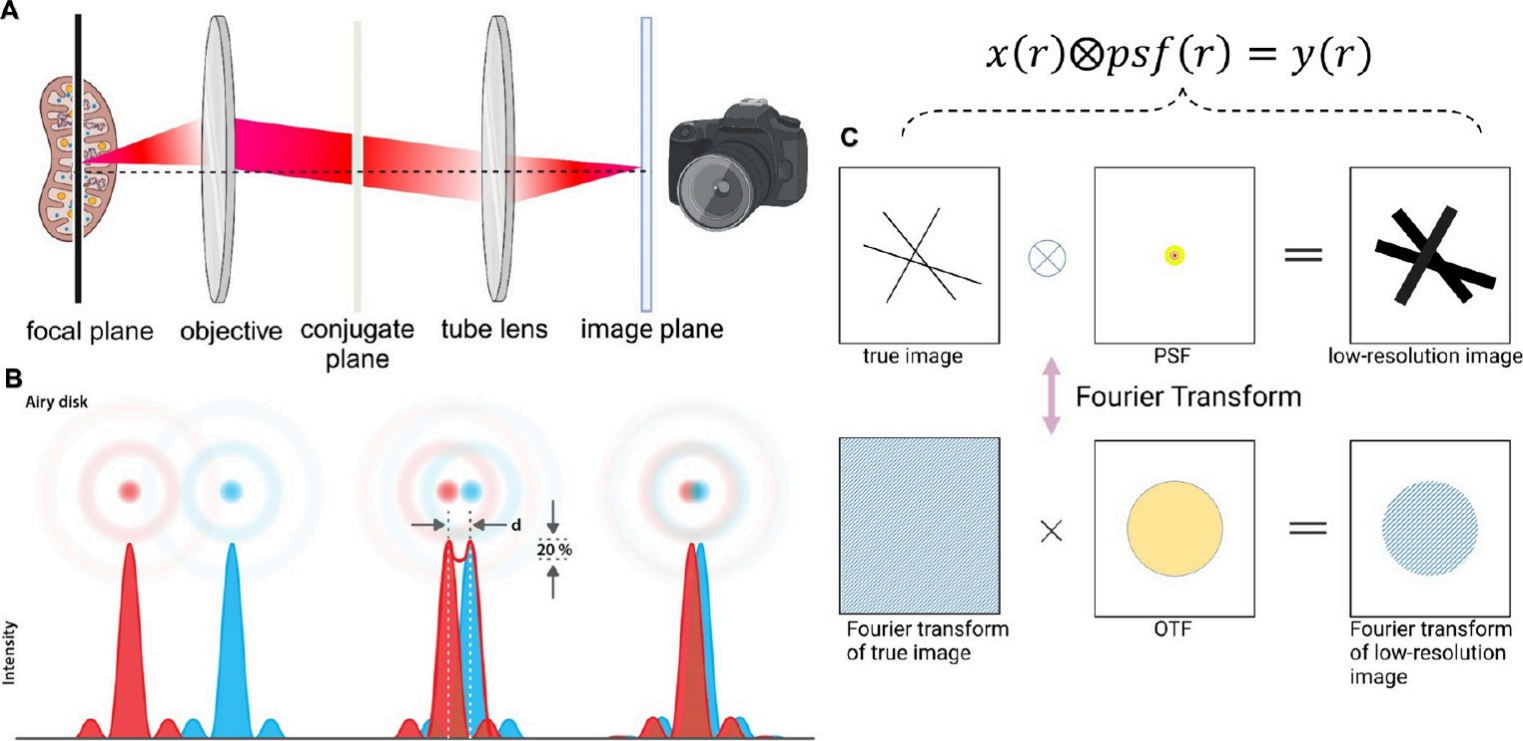
Key concepts of optical imaging. (A) An abstract representation of a fluorescence microscope. The fluorescence emitted from the focal plane is collected by a camera, such as a CCD, at the image plane to form an image. (B) Overlap of the Airy functions. Rayleigh determined that the human eye can resolve a ± 20% decrease in intensity, which corresponds to the overlap of the Airy disk of one Airy function with the first minimum of another. On the left, two resolved Airy functions are shown. On the right, two unresolved Airy functions are depicted. The middle image represents two Airy functions separated by the Rayleigh limit. (C) Spatial and frequency domain representations of the imaging process. (Top) The imaging process can be modeled as the convolution of the sample with the PSF, shown here in a 2D plane. CCD: charge coupled device. (B) is reprinted with permission from ref (34). Copyright 2018 IOP Publishing Ltd., licensed under a Creative Common Attribution (CC BY) 3.0 license. (A) and (C) were created in https://BioRender.com.

As illustrated in Figure 2(A), the core of the microscope consists of two lenses (an objective lens and a tube lens) separated by their focal lengths. The intermediate (virtual) plane between these lenses is referred to as the conjugate plane. The focal plane, where the sample is positioned, is located at the front focal plane of the objective lens, while the image plane, where the camera is placed, lies at the back focal plane of the tube lens. The system is termed a 4f system because the focal plane and image plane are separated by four focal lengths.

Due to the wave nature of light, an infinitesimal point source in the focal plane is imaged as a blurred spot in the image plane, known as an Airy disk for a circular aperture. Figure 2(B) shows two Airy disk functions. Generally, the spatial distribution of this spot is termed the point spread function (PSF).

An imaging target can be modeled as a collection of infinitesimal points, which, after passing through the microscope, become superimposed PSFs. This superposition resulted in a blurred image.

Mathematically, this process is described as
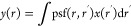
1Here, *r* represents spatial coordinates, *x* is the unknown sample with a fluorescence distribution in the focal plane, and *y* is the resulting blurred image in the image plane. The imaging process is characterized by the PSF. We assume a shift-invariant PSF, meaning its shape remains unchanged with spatial shifts, which is valid for most optical microscopes. Thus, eq 1 is a convolution process, where ⊗ denotes convolution. This is depicted in Figure 2(C), which shows initially sharp lines becoming blurred after imaging.

In practice, *y* will be reconstructed into a digital image that can be represented as a 2D (or three-dimensional) vector. Each element of the vector corresponds to an image pixel.

Therefore, all continuous functions in eq 1 are replaced with discrete vectors and , and the imaging equation transitions from an integral to a discrete convolution. Additionally, noise will inevitably appear, leading to the complete imaging equation:
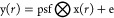
2where e represents the noise. Note that although noise is represented additively here, it can take more complex forms, such as Poisson noise in photon-limited cases, and can be even more nuanced in SR fluorescence microscopy.^35^ The detailed derivation can be found in the Fourier optics textbook.^36^ Additionally, we provide further details in the “Details of Imaging Process” section of the Supporting Information.

It is well-known that the Rayleigh resolution limit corresponds to the overlap of two Airy disks (Figure 2(B)). It is calculated that a ±20% decrease in intensity can barely be resolved by the human eye. This occurs when the center of one Airy function overlaps with the first minimum of the second Airy function (Figure 2(B) middle). However, this is an empirical criterion. Another resolution definition is the full width at half-maximum (FWHM) of the PSF. However, it is challenging to identify independent PSFs in a single fluorescence image. This poses practical difficulties in defining the SR.

**Figure 3:**
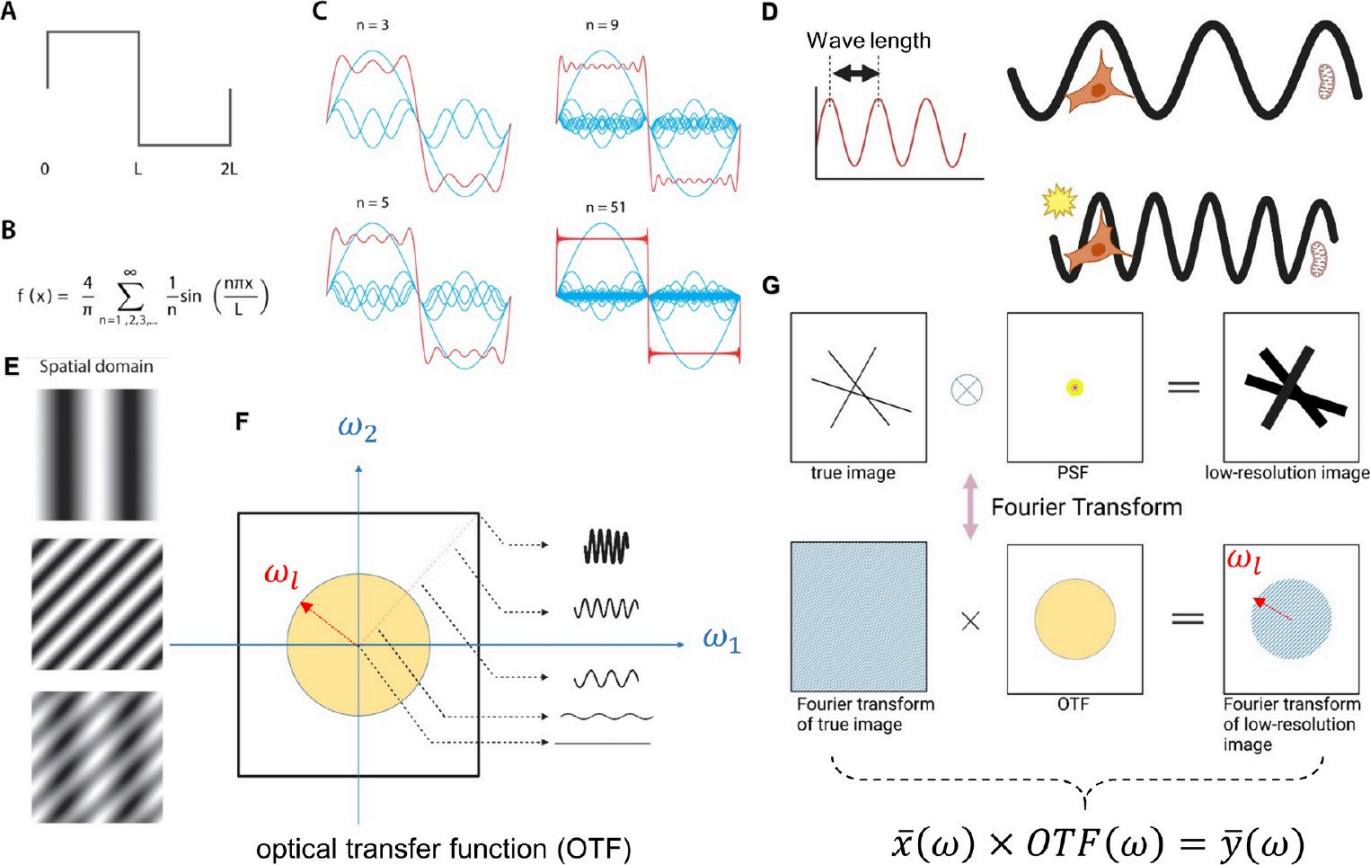
Imaging in the perspective of the Fourier domain. (A–C) Signal decomposition in Fourier theory. (A) A step function representing an arbitrary signal. (B) This complex shape can be approximated by a sum of sine functions. (C) To approximate the original signal faithfully, a large number of sinusoidal signals (blue) need to be summed (red). (D) (Left) Wavelength of a sine wave. (Right) High-frequency (short-wavelength) waves correspond to higher resolution. Intuitively, high-frequency waves are less able to bypass small objects, thereby resolving smaller features. (E) Fourier transforms of two-dimensional images. The top and middle show two 2D sine waves with different orientations and frequencies, which can be combined to form the image shown at the bottom. (F) Two-dimensional frequency spectrum and the optical transfer function (OTF). In the 2D frequency domain, sine waves are represented by their frequency and orientation (only 1D components are shown here, so orientation is not explicitly indicated). Higher frequencies are located farther from the origin. The OTF is a function limited to low-frequency components (yellow circle) with a cutoff frequency of ω_*l*_ (red arrow). (G) Spatial and frequency domain representations of the imaging process. (Bottom) In the frequency domain, the convolution corresponds to multiplication. The sample’s frequency domain signal is multiplied by the OTF, leading to the loss of high-frequency components beyond the cutoff frequency ω_*l*_. (A–C, E) are reprinted with permission from ref (34). Copyright 2018 IOP Publishing Ltd., licensed under a Creative Common Attribution (CC BY) 3.0 license. (D, F, G) were created in https://BioRender.com.

On the other hand, the core of “Fourier optics” resides in the Fourier transform, which decomposes signals into a weighted sum of sinusoidal components. This provides a frequency-domain definition of the imaging resolution. Figure 3(A–C) illustrate how a step function is approximated using an infinite series of sinusoidal functions . Notably, as *n* increases, the sharp edges of the superimposed signal become increasingly clear. This indicates that a larger *n* corresponds to a sharper signal.

As shown on the left side of Figure 3(D), the distance between two peaks of a sine wave is called the wavelength λ, and the reciprocal of the wavelength is the frequency . A larger *n* also implies a higher-frequency signal, which corresponds to a sharper signal or, in other words, a higher resolution. Although there are complex physical implications behind this, it can be intuitively understood as follows: longer-wavelength (lower-frequency) sine waves can more easily bypass large objects, whereas shorter-wavelength (higher-frequency) waves are more likely to interact with smaller objects, thereby resolving underlying structures (Figure 3(D)).

The same principle can be easily extended to 2D images, which are essentially a superposition of spatial frequencies with varying orientations. As shown in Figure 3(E), the top and middle images represent two 2D sine waves with different orientations and frequencies. Their superposition (bottom) results in a more complex 2D image.

These 2D sine waves can be represented in a 2D frequency-domain image based on their orientation and frequency. As illustrated in Figure 3(F) (note that the orientation is not shown in the figure; therefore, 1D sine waves are used to represent 2D sine waves with a specific orientation), the origin point represents a plane wave with no intensity variation. Along a given line, 2D sine waves with higher frequencies are located farther from the origin. Just like in 1D, 2D sine waves with higher frequencies correspond to higher image resolution.

Therefore, the Fourier transform links the resolution to the frequency domain: the resolution of an image is defined as the reciprocal of the highest frequency component in its spectrum. This frequency-domain analysis allows resolution to be defined objectively, independent of specific imaging details, which is an advantage over other definitions.^37^

This allows the imaging process to be understood from a frequency-domain perspective. The Fourier transform of the PSF is known as the Optical Transfer Function (OTF). The OTF describes how different frequency components of the true image are modulated as they pass through the optical system. The most significant property of the OTF is that it is band-limited (Figure 3(F), yellow circle), meaning that only 2D frequency components with frequencies below the cutoff frequency ω_*l*_ (Figure 3(F), red arrow) can pass through the imaging system. This ultimately limits the imaging resolution.

Mathematically, in Fourier space, convolution becomes a multiplication, simplifying the imaging equation to
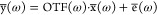
3where ω is frequency variable. The high-frequency components of the true image beyond the cutoff frequencies ω_*l*_ are filtered out by the OTF, making the microscope effectively act as a low-pass filter (Figure 3(G)). This low-pass filtering effect fundamentally limits the resolution. As described by Abbe’s criterion, the diffraction limit resolution *d* is
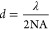
4where NA is the numerical aperture. For biological research, this limit typically corresponds to ∼250 nm of lateral resolution and ∼750 nm of axial resolution.

On the other hand, the OTF is three-dimensional. Another property of the OTF is the “missing cone”, meaning classical optical microscopy cannot distinct in-focus light and out-of-focus photons. More detail is provided in the Supporting Information under “Details of Imaging Process”.

### Computational Super-Resolution as an Ill-Posed Problem

The resolution limit defines the SR problem as the recovery of high-frequency components of the true image beyond the cutoff frequency. This process is often referred to as “out-of-band extrapolation”. The concept of SR was first introduced by Toraldo di Francia in 1952,^38^ who described it as enhancing the angular resolution of an optical system beyond the diffraction limit. This aligns with the modern definition of the SR.

We define computational super-resolution (CSR) as follows: given a low-resolution image y and the PSF in the imaging eq 2, CSR aims to derive or approximate the true high-resolution image using specific algorithms. In this Review, x is referred to as the *true image* or *true signal*, y as the (imaging) *data*, and the algorithm’s output as the *model image* or *model signal*, denoted as x*.

In the literature, CSR is frequently conflated with terms such as deconvolution and even deblurring. While CSR inherently involves countering the effects of convolution, it specifically focuses on out-of-band extrapolation. Note that, by this definition, filling the missing cone of the OTF, that is, enhancing the optical sectioning capability of the microscope, is also a form of SR. In contrast, deconvolution may also refer to normalizing the contrast within the cutoff frequency due to OTF attenuation, which aligns with the concept of deblurring, and resolving previously unresolved high-frequency information due to low signal-to-noise contrast. In this Review, we define deconvolution as focusing on out-of-band extrapolation and deblurring as enhanced contrast within the OTF.

**Figure 4:**
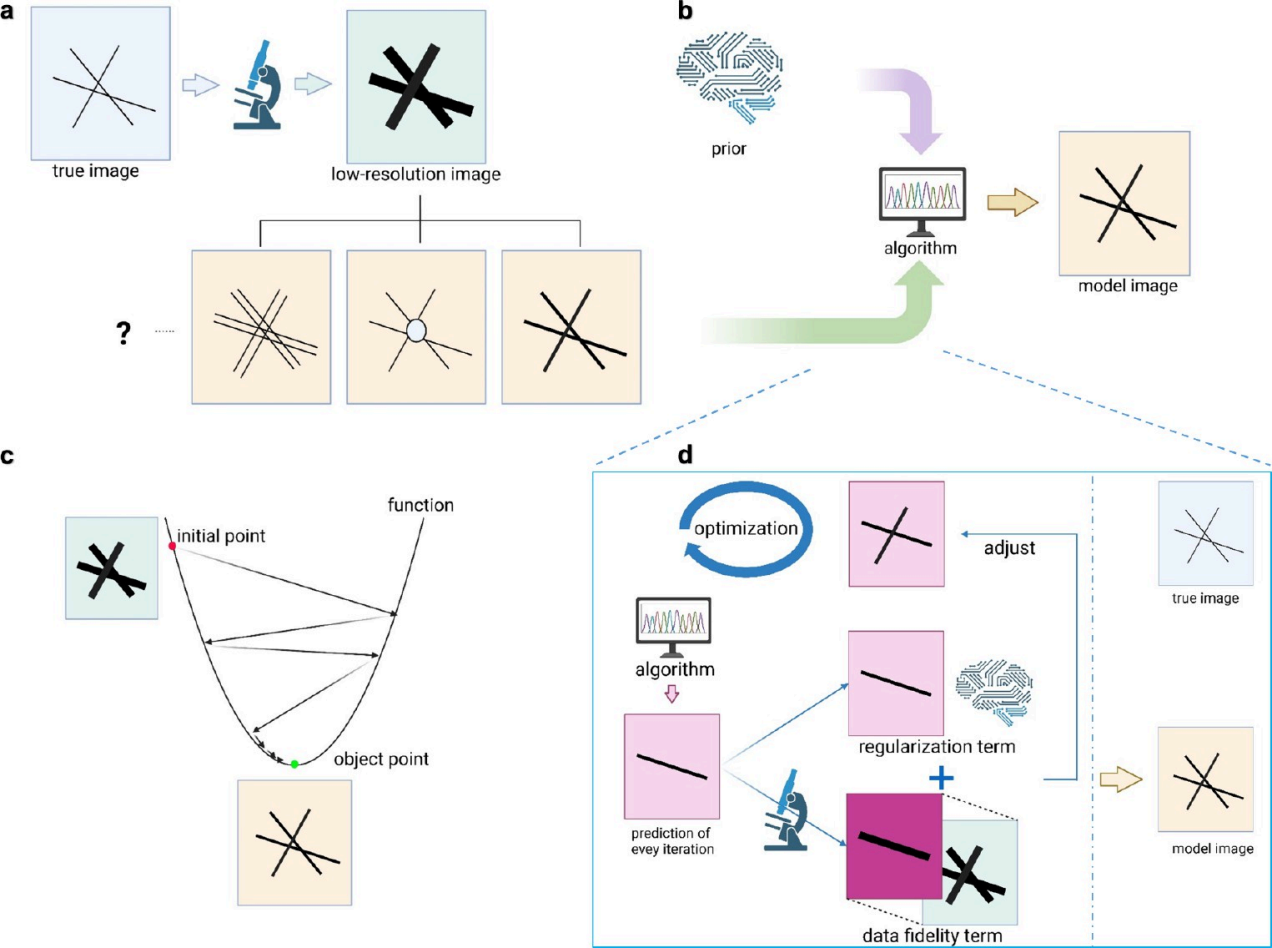
The framework of computational SR (CSR). (a) CSR as an ill-posed problem. Due to the low-pass filtering effect of microscopes, the true image is degraded into a low-resolution image. The loss of high-frequency information results in infinitely many possible high-resolution images corresponding to the same low-resolution image. (b) Solving CSR with priors. The introduction of priors helps address the ill-posed nature of CSR by constraining the set of possible solutions. Through specific algorithms, these priors enable the recovery of a high-resolution image that closely approximates the true image. (c) Iterative optimization for solving the CSR. This panel illustrates the iterative process of solving an optimization problem. The target is to minimize an objective function (denoted by the green dot as the object point). Starting from an initial guess (red dot, often the low-resolution image), the optimization algorithm takes steps toward the minimum point according to a specified rule, such as gradient descent. After several iterations, the algorithm converges to the minimum point. (d) The basic framework of CSR. The CSR framework involves defining a prior (regularization term), determining the data fidelity term, and solving the corresponding objective function through optimization. This figure was created in https://BioRender.com.

Since the OTF limits high-frequency information beyond the cutoff frequency, an infinite number of possible high-frequency models could correspond to the same low-resolution data. As shown in Figure 4(a), after passing through the microscope imaging system, fine lines originally become blurred and thicker and an infinite number of possible images can result in the same thick-line image when processed by the imaging system. Furthermore, we do not directly observe the low-resolution data; instead, it is affected by noise and aberrations.

This reveals the central challenge of CSR: it is an ill-posed problem. In mathematics, a problem is considered well-posed^39^ if it satisfies three conditions:

1. A solution exists.
2. The solution is unique.
3. The solution process is stable (if the reader wants a formal mathematical definition, please see ref (40)).

An ill-posed problem fails to satisfy at least one of these conditions. In the case of the CSR, the last two conditions are not met.

### Linear Algebra View of Computational Super-Resolution

The core idea to solve ill-posed problems is to impose a “priori” constraint, allowing a stable algorithm to select a unique image from all those that are consistent with the data. This process is illustrated in Figure 4(b). This has a clear mathematical meaning in linear algebra. The imaging eq 2, that is, the convolution process, can be represented as matrix multiplication:
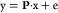
5where **P** is the PSF matrix. (Readers unfamiliar with this can refer to the Supporting Information section “Convolution as Matrix Multiplication” for the derivation.) In this context, the CSR problem transforms into a least-squares (LS) problem:
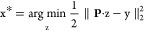
6Here ∥•∥_2_ is the *l*_2_ norm, with vector z as the independent variable. The term “arg min” means obtaining the value of the independent variable that minimizes the function on the right (*l*_2_ norm takes its minimum value). In mathematics, finding this extremum is termed solving an optimization problem. If the matrix **P** is full rank, it allows for a solution named direct inversion:
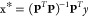
7However, two problems arise: first, matrix **P** is not full rank. It has a nontrivial null space, containing infinitely many vectors that, when added to x*, still satisfy the equation. This corresponds to the previous issue: due to the loss of high-frequency information, there are infinitely many possible high-resolution images that map to the same low-resolution image. Second, **P** is related to submatrices of the Fourier matrix, which have very large condition numbers.^41^ Consequently, the presence of noise exacerbates the difficulty of solving the LS problem, as even small perturbations in the observed data can lead to significant deviations in the solution.

In linear algebra, solving this (named under-determined) equation typically requires “regularization”. Generally, regularization is a process that simplifies the solution of a problem by introducing constraints or additional information. Specifically, regularization introduces an additional penalty term to the LS problem:
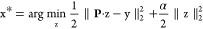
8where  is the penalty term and α > 0 is the penalty parameter. This modification ensures that **P**^T^**P** + α**I** is symmetric and positively definite, providing a unique solution:
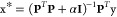
9

This regularized approach also helps stabilize the solution and address the issues arising from noise.

### The Basic Function in Computational Super-Resolution

Beyond the source of prior information, a unified mathematical framework is needed to model them. Without loss of generality, we define a prior as a constraint modeled by a penalty function *R*(x), which measures the agreement of the image with the priors: the lower *R*(x), the better the agreement. For example, true high-resolution images tend to be noise-free, so the prior reason can be that the pixel values in the model image should be smooth. The  norm of a differential operator is a good choice to impose this constraint, a method known as classic Tikhonov regularization.^42^ The smoother the image, the smaller the penalty function  should be.

However, priors alone are not sufficient (as the smoothest possible image, for example, would be a constant image). The model image also needs to be constrained by the observed data. The objective is to find a model image x* that aligns with the observed low-resolution data y and satisfies the priors. This leads to a constrained optimization problem:
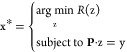
10However, due to the presence of noise, ensuring the model image exactly matches y would be misleading. Therefore, some expected discrepancy between the model and the observed data is considered acceptable. We set η(e) as the error level according to the real noise e, and modify the optimization problem to
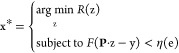
11Here, *F*(**P**·z-y) ≔ *F*(z, y) represents the expected discrepancy, quantifying the fidelity of the model to the observed data. Ideally, the result depends on the noise model, but for simplicity, it is often taken as the square of the  norm.

Using the Lagrange multiplier method (see Supporting Information on the “Lagrange Multiplier Method”), the constrained optimization problem can be reformulated as an unconstrained optimization problem:
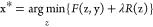
12In this Review, we refer to this optimization problem as the “basic function”, with *F*(z, y) representing the “data fidelity” term and *R*(z) representing the “regularization” term. Here, λ ≥ 0 is the Lagrange multiplier, which balances the data fidelity and prior adherence. Under suitable conditions, the solutions to both optimization eqs 11 and 12are equivalent. Notably, eq 8 is a special case of this basic function.

Unlike direct inversion, the basic function in CSR is typically complex, making a direct analytical solution infeasible. Instead, iterative algorithms are employed (Figure 4(c)). The exact shape of the basic function is often unknown; in this example, it is represented as a parabolic curve with a minimum point. The algorithm begins at an initial point, usually the low-resolution image, and iteratively moves closer to the minimum point by following optimization rules. If conditions are suitable, such as when the objective function is convex, the algorithm will converge to the minimum point after a finite number of iterations. (Readers unfamiliar with convex and nonconvex optimization can refer to the Supporting Information on “Convex and Non-Convex Optimization”).

The process for solving the CSR using the basic function is summarized in Figure 4(d). First, the prior (regularization term) and the data fidelity term are determined. Then, an iterative algorithm is applied. In each iteration, the algorithm generates a predicted image, compares it to the observed data through the forward process, and evaluates its consistency with the priors. The error feedback is used to adjust the predicted image according to specific optimization rules. While this explanation is intuitive, rigorous mathematical derivations can validate this process in concrete implementations.

The core of CSR is the use of priors. There are alternative ways to model priors using different mathematical frameworks. One widely used approach treats the unknown high-resolution image as a random variable, with the prior represented as its probability distribution; this is known as the Bayesian framework. Further details on this modeling approach are provided in the Supporting Information section titled “Bayesian Views of Priors” for the readers’ reference.

The choice of priors determines the various methods utilized in CSR. Given CSR’s close integration with microscopy, the proposal of both common and specific priors has significantly shaped its developmental trajectory. In the following sections, we will trace the fascinating history of CSR’s evolution, organized around its key methodological advancements.

## Early Explorations

**Figure 5:**
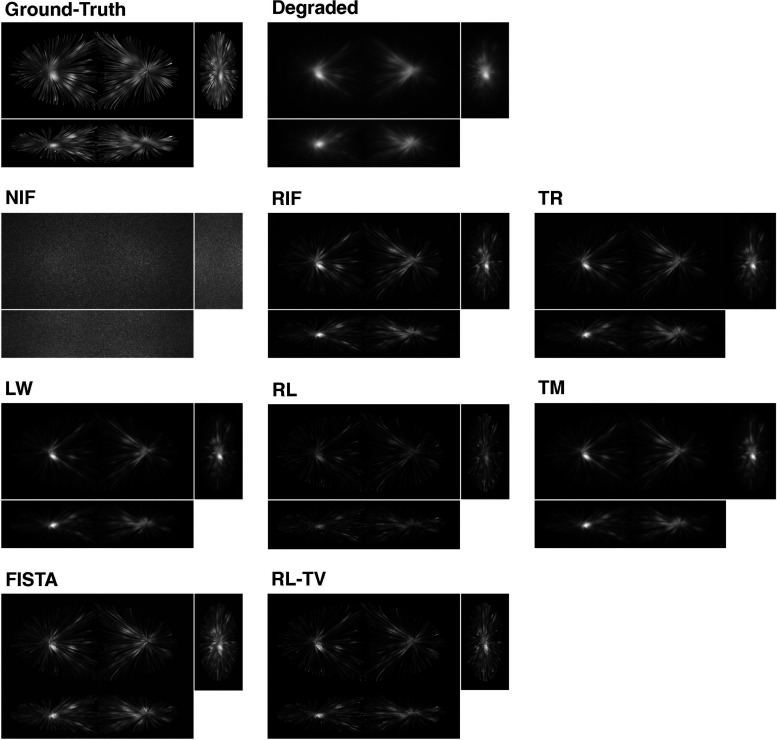
Orthogonal sections of the maximum intensity projection (MIP) of a degraded 3D synthetic volume after deconvolution using various algorithms. From top left to bottom right: ground-truth volume, degraded volume (after convolution with PSF and simulate noise), naïve inverse filter (NIF), regularized inverse Filter (RIF), Tikhonov regularization (TR), Landweber iteration (LW), Richardson–Lucy (RL), Tikhonov–Miller (TM), Fast Iterative Shrinkage-Thresholding Algorithm (FISTA, *l*_1_ minimization), and Richardson–Lucy with total variation (RL-TV). A non-negativity constraint was used for all algorithms. The setting of the optimal parameters for each deconvolution algorithm was performed through visual assessment. Reprinted from with permission from ref (54). Copyright 2017 Elsevier.

We will first review the development of CSR from the mid-20th century to the 1980s, a period before the emergence of SR fluorescence microscopy, highlighting key methods and analyzing their limitations. Figure 5 shows the performance of several algorithms discussed in this section on simulated images.

### Inverse Filters

In the early 20th century, the theory of linear systems was established. Early attempts to solve CSR treated it as a linear signal recovery problem.

Without additional information, the only way to recover input *x* from output *y* is to approximate *x* as a linear superposition of *y*:
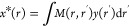
13This linear *solver* or *estimator* is characterized by the function *M*(*r*, *r*′), in Fourier space:
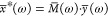
14Here, *M̅* is termed a filter in signal processing. Ideally, M̅ = [OTF] ^–1^, so such processing is called an inverse filter.

The simplest inverse filter is the naïve inverse:
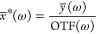
15However, this naïve inverse approach has two major problems. First, the OTF is band-limited, meaning that frequencies beyond the cutoff are zero. This makes direct inversion impossible for these frequencies. A workaround is to constrain the inverse operation to within the cutoff frequency. Second, and more critically, noise in the data is significantly amplified in frequency regions where the OTF(ω) is small, especially near the cutoff frequency. As a result, the naïve inverse fails to preserve meaningful image information. The performance of the naïve inverse filter is shown in Figure 5 (NIF), where the result is full of noise.

The classical Wiener filter^43^ improves noise suppression by providing the best linear estimate in the least-squares sense (eq 6). Setting *M̅* as the Wiener filter, the result is
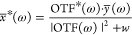
16where OTF* is the conjugate of the OTF, |OTF|^2^ is power spectrum of OTF, and *w* is the Wiener parameter. This method is also known as Wiener deconvolution. If the noise is assumed to be white, then an optimal Wiener filter can be derived. Wiener deconvolution is equivalent to Tikhonov regularization, as shown in Figure 5 (TR).

To further suppress noise, additional linear regularization can be applied, transforming the least-squares problem into a regularized least-squares problem:
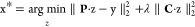
17where **C** a is transform matrix. The inverse filter in this case is
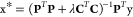
18Here, **C**^**T**^**C** acts as a filter. Equation 9 is a special case of this formulation.

These algorithms are often used to suppress noise for the initial recovery of x. In SIM reconstruction, Wiener deconvolution is a standard method.^9^ More precisely, inverse filters are used to correct attenuation within the cutoff frequency and improve contrast, similar to how the human eye enhances resolution; this is essentially what deblurring means.

However, the fundamental flaw of linear algorithms is that they can only provide information based on their input. Since the data does not contain any high-frequency information, the linear superposition of data still cannot generate new information. Consequently, inverse filters are not effective in recovering the out-of-band spectrum. Thus, more advanced approaches are needed.

### Analytic Continuation

When Francia introduced the concept of super-resolution in 1952, he realized, “*achieving image resolution greater than the diffraction limit lies in the ambiguity which generally attends the extension of a complex spectrum which is known over a finite interval only”*.^38^

However, in 1964, Harris proposed a method based on a straightforward prior: the imaging object has a finite spatial extent.^44^ Under this assumption, it can be proven that ambiguity does not exist. In such cases, the true image spectrum is an entire analytic function and is uniquely determined by the part of the spectrum transmitted by the optical system. Therefore, the required out-of-band extrapolation— SR—can be performed, at least in theory. This concept is known as “analytical continuation”. A precise definition is provided in the Supporting Information section on the “Analytical Continuation of CSR”. However, in practice, noise renders this theory ineffective.

### Prolate Spheroidal Wave Functions

Analytic continuation inspired the theoretical foundation for out-of-band extrapolation, with the extent of extrapolation determined by a set of special functions known as prolate spheroidal wave functions (PSWFs),^45^ denoted as ψ_*n*_(*c*, *r*), where c is the space–bandwidth parameter determined by ω_*l*_. Detailed mathematical explanations are provided in the Supporting Information section titled “Mathematical Meaning of PSWFs”.

Under the assumption of a finite spatial extent and a box-like OTF (no attenuation within the cutoff frequency, which can also be achieved through deblurring), the true image can be represented as^46^
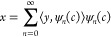
19This equation indicates that the true image is uniquely determined by the data, aligning with the theory of the analytic continuation. In principle, the coefficients ⟨*y*, ψ_*n*_(*c*)⟩ can be derived from *y* using PSWFs and then used to reconstruct true image. However, as *n* increases, the coefficient approaches zero and, after a certain point, falls below the noise level. The effective number of recoverable coefficients depends on the SNR.

Conversely, we can approximate the true image using a truncated expansion according to the effective number of coefficients. The goal is for the approximated image to have a resolution higher than the cutoff frequency. Historically, PSWFs provided theoretical insight by demonstrating that even though infinite extrapolation is not feasible, limited SR improvement is possible. This redefined CSR as an extrapolation problem, aiming to recover the out-of-band spectrum given the noisy spectrum within the cutoff.

### Gerchberg–Papoulis Algorithm

A simple iterative algorithm for the extrapolation problem, based on the finite spatial extent prior, is the Gerchberg–Papoulis (GP) algorithm, proposed in the 1970s.^47,48^ We defer the details of the algorithm to the Supporting Information section titled “Gerchberg–Papoulis Algorithm”.

In the absence of noise, this algorithm theoretically converges to the correct solution,^49^ as the uniqueness implied by analytic continuation guarantees. However, in the presence of noise, the algorithm risks overfitting. Often, early termination yields better results, while adding regularization can further suppress noise. Despite its limitations, the GP algorithm was a pioneering method and its iterative approach inspired future developments in CSR.

In the 1970s and 1980s, it seemed that the CSR problem had been addressed, from theory to algorithm, based on the finite spatial extent prior. However, in practice, it was found that the increase in resolution was very limited, falling short of achieving SR. The main reason for this shortfall is that the prior’s modeling was too simple, leading to a limited constraint effect. As noted in the famous textbook *Introduction to Fourier Optics*, “*While the fundamental mathematical principles are most easily stated in terms of analytic continuation...this method has not proven successful in practice*”.^36^

From this point on, CSR gradually transitioned from being an extrapolation problem to one solved through optimization-based methods.

### Iterative Deconvolution

For historical reasons, CSR methods based on iterative algorithms are often referred to as “deconvolution”, and the field is also known as deconvolution microscopy.

The first type of method replaces direct inversion with gradient descent. Directly applying gradient descent to solve the least-squares (LS) problem in eq 6 yields the Landweber iteration (LW):^50^
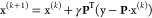
20where γ is the step size. Occasionally, to ensure non-negativity, additional calculations max{*x*^(*k*+1)^, 0 } are performed in each step (Figure 5, LW). Similar to the GP algorithm, LW can suffer from overfitting if the iteration count is too high. At first glance, LW does not seem to provide additional priors compared with the inverse filter. However, the iterative process itself bypasses the divided by zero operation and serves as a form of constraint. This helps ensure that the solution is closer to the true solution, which also justifies the use of deconvolution methods. Later studies demonstrated that the number of iterations can serve as a regularization parameter,^51^ and it was further proved that GP is a special case of LW.^16^

LW demonstrated the ability to enhance optical sectioning in conventional microscopy. In 1983, Agard and Sedat used LW to enhance the resolution of the observed three-dimensional distribution of nuclear chromosomes.^52^ Similarly, applying gradient descent to solve a regularized LS problem leads to the Tikhonov–Miller iteration^53^ (Figure 5, TM):
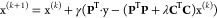
21where λ is a regularization parameter and **C**^**T**^**C** represents a transform. Although LW and TM originated in earlier decades, their application in image processing became practical much later due to computational limitations. When the transform matrix **C** is related to the properties of the image, a popular choice is smoothness, implying that adjacent image pixels tend to have similar values. Thus, **C** can be the discretization of a differential operator such as the Laplacian operator.

### Richardson–Lucy Deconvolution

Another class of iterative SR algorithms is based on assumptions about the probabilistic distribution of random variables. Richardson–Lucy deconvolution (RL) proposed by Richardson (1972)^55^ and Lucy (1974)^56^ has played a pivotal role in CSR. Its formulation is as follows:
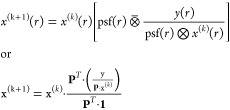
22where ⊗̅ denotes the transpose of convolution and **1** represents a vector of ones with the same size as vector y. Sometimes, the numerator and denominator in the RL formulation are referred to as the “inverse projector” and the “projector”, respectively. The RL method assumes that noise follows a Poisson distribution, which is a fundamental characteristic of photon detection. The detailed derivation of RL can be found in the Supporting Information section titled “Richardson-Lucy Deconvolution Detail”.

RL exhibits SR capabilities on simulated data (Figure 5, RL). Unlike other iterative algorithms, RL has been shown in many studies to achieve SR, particularly in radio astronomy.^57,58^ Specifically, RL has demonstrated the ability to surpass the Rayleigh criterion in separating double stars in astronomical imaging. As RL lacks an explicit regularization term, it must possess some inherent regularization properties. Like other iterative algorithms, the number of iterations itself acts as a form of regularization.^59^ At the same time, RL naturally ensures non-negativity and preserves the total photon count, aligning with the physical meaning of the PSF.^60^

However, RL also has its unique weaknesses. It has been observed in practice that RL is sensitive to noise and prone to produce pattern-like artifacts, such as the ringing effect^61^ and sparsity effect.^62^ The understanding of these artifacts is still unsatisfactory, and they limit the practical application of RL in achieving SR. For example, RL has been proven to be infeasible for solar system imaging.^63^ Some studies have introduced additional regularization terms to suppress noise,^64^ but these efforts have achieved only limited success. It was not until recent developments, which combined several methods, that significant breakthroughs were achieved,^10^ as will be discussed later. Another problem with the RL is its slow convergence rate. Recently, modifications to the inverse convolution operator with an unmatched back projector have achieved a speedup of several thousand-fold.^65^

Unfortunately, first proposed as an engineering approach, the mechanisms by which RL achieves super-resolution and exhibits its other demonstrated characteristics are still not fully understand. In fact, it was noted in 2015 that “*The understanding of the RL algorithm and proper stopping is still an open issue and has not been solved satisfactorily*”.^35^ Despite these challenges, RL remains one of the most commonly used deconvolution algorithms in biological imaging. It is frequently employed in confocal and wide-field microscopy to deblur images and enhance contrast. As will be shown in the applications section, RL has recently been used for fluctuation-based SR fluorescence microscopy,^12^ light sheet microscopy,^65^ and spatial transcriptomics.^14^

### Summary on Early Explorations

In this section, we present the key achievements and limitations of early CSR. The two main approaches explored were analytical continuation theory and iterative deconvolution. To be honest, these methods have achieved only limited progress. Perhaps the most appropriate annotation for this comes from the autobiography of STED inventor and Nobel laureate Stefan W. Hell, who stated:^66^ “[before invention of STED] *it was widely believed that the route towards higher resolution in the far-field was data processing, which typically required some assumptions about the object...Yet, none of these concepts were practical, or got beyond a factor of two*.”

This underscores the challenges faced by early CSR methods in achieving substantial improvements in resolution. On the other hand, these pioneering efforts have left a rich legacy and paved the way for the next stage of CSR: model-based CSR.

## Model-Based CSR

A major flaw of early iterative algorithms such as LW, TM, and RL is their lack of regularization terms or reliance on linear regularization. Since the 1990s, model-based CSR has evolved significantly, with a focus on nonlinear regularizations that enhance prior modeling and address the limitations of early deconvolution methods.

In this section, we chronologically introduce milestone works in model-based CSR, focusing on sparsity priors and leading up to recent breakthroughs such as sparse-deconvolution.

### Maximal Entropy Deconvolution

A classical nonlinear regularization method is maximal entropy deconvolution (ME),^67^ expressed as
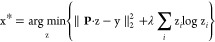
23

Here, the regularization term represents the maximum entropy regularization term. Entropy, a physical concept measuring system disorder, is used in this context. The fundamental idea behind maximum entropy regularization is to select the solution that is the least biased or assumes the least amount of additional information beyond what is provided by the data. For fluorescence imaging, maximum entropy assumes that the most probable distribution of fluorescence intensity is the smoothest distribution that still matches the observed data.

Like the RL, maximum entropy regularization preserves positivity. It is widely used in microscopy to suppress noise while preserving the sample structure. For instance, Arigovindan et al. (2013) proposed ER-Decon (entropy-regularized deconvolution) to enhance low-dose wide-field fluorescence imaging, enabling high-resolution in vivo imaging under extremely low phototoxicity conditions.^68^

### Total Variation

While smoothness suppresses noise, it also damages high-frequency information, conflicting with CSR objectives. A more refined approach utilizes nonsmooth regularizations. The most classic of these is total variation (TV), introduced in the 1990s.^69^ Originally proposed from a functional space and partial differential perspective,^70^ the discrete form of TV is
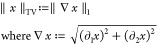
24where ∂_*i*_ represents the differential operator in direction *i* (the row and column orientations of the image).

When used as a regularization term, the TV-regularized problem becomes
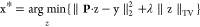
25

TV assumes that the image consists of piecewise-constant regions separated by sharp boundaries. In biological imaging, this means that TV tends to reconstruct a sharp fluorescence image by assuming that the true image has relatively smooth regions (e.g., cytoplasm) and sharp boundaries (e.g., cell membranes). With this prior, TV is effective for preserving edges while reducing noise.

TV was first applied to the CSR for deblurring and denoising. For instance, it has been combined with RL to enhance confocal microscopy deconvolution^71^ (also shown in Figure 4, RL-TV). However, TV’s preference for piecewise-constant representations can lead to “cartoon-like” effects in reconstructions. To address these limitations, advanced versions such as higher-order TV have been developed.^72^

### Multi-Resolution Analysis

Biological images capture resolution information across multiple scales such as organelles, protein complexes, and molecular structures. This multiscale nature can itself serve as a prior. Multiresolution analysis (MRA), originating in the late 1980s with wavelets,^73^ is a method to analyze an image’s features at various resolutions.

A major tool of MRA is the discrete wavelet transform (DWT), which relies on recursively decomposing an image into low- and high-frequency components as follows:
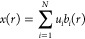
26where {*b*_*i*_(*r*)}_*i*=1···*N*_ are the wavelet functions and {*u*_*i*_}_*i*=1···*N*_ are wavelet coefficients. A wavelet function is a mathematical function that resembles a small wave. Unlike traditional sine waves used in Fourier analysis, wavelets are localized in both space and frequency, allowing them to capture fine details of a signal in specific regions. (The detailed mathematical definition is beyond the scope of this Review and will not be covered here.) The key property of wavelets, orthogonality, ensures that each level of detail is independent.

DWT can analyze images at different levels of resolution, similar to zooming in and out of a picture. In CSR, the high-resolution image contains more frequency components in the wavelet domain compared with the low-resolution image. Thus, CSR can be reformulated as a problem of reconstructing the missing high-frequency components. This is achieved by transforming the low-resolution data into the wavelet domain and iteratively refining the detail coefficients.

However, DWT is primarily used for denoising in microscopy, as noise often has small coefficients in the wavelet domain due to its incoherent structure. By applying thresholding to remove these small coefficients, the signal can be separated from the noise. When combined with iterative algorithms, this approach can improve the resolution. For example, Boutet de Monvel et al. applied wavelet denoising to confocal microscopy and combined it with maximum entropy regularization, achieving better resolution in biological applications of imaging the organ of hearing.^74^

Framelets and curvelets extend the capabilities of wavelets, allowing for more flexible signal decomposition, especially in handling geometric features, such as edges and curves, in images. Assume high-resolution fluorescence images often exhibit characteristics such as edge continuity and cross-edge sparsity,^75^ it is proposed to use framelet and curvelet transforms as the prior:
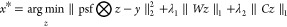
27

Here, *W* denotes the framelet transform and *C* denotes the curvelet transform. The authors claim that this framework may improve the SNR and can provide up to a 2-fold increase in fidelity-ensured resolution improvement. However, this assertion also critically depends on the assumption of edge continuity and cross-edge sparsity.

### Sparsity Prior

We do not delve into the above nonlinear regularizations in detail because, in CSR, they only show significance when combined with a sparsity prior. Sparsity prior, or sparse regularization—particularly convex approaches based on  norm minimization (well-known in Compressed Sensing theory)—is a cornerstone for addressing ill-posed problems and has become foundational also in CSR. In the following sections, we will introduce CSR based on the sparsity prior. We include only the necessary concepts, deferring precise mathematical concepts to the Supporting Information section titled “Mathematical Concepts in Sparsity Prior”.

In general, a sparsity prior refers to the knowledge that the real-world data can be represented as a combination of only a few significant terms. Sparsity is closely tied to the famous Razor principle.

In biology, sparsity is very common. For example, the retina encodes images using a sparse set of ganglion cells; the olfactory system utilizes a sparse set of receptor proteins to detect a wide variety of odors; and within a given cell, only a sparse number of genes exhibit notable expression. Most importantly, designated molecules are sparsely labeled and excited to produce highly contrasted and sensitive fluorescence images as opposed to the comprehensive view provided by an electron micrograph.

**Figure 6:**
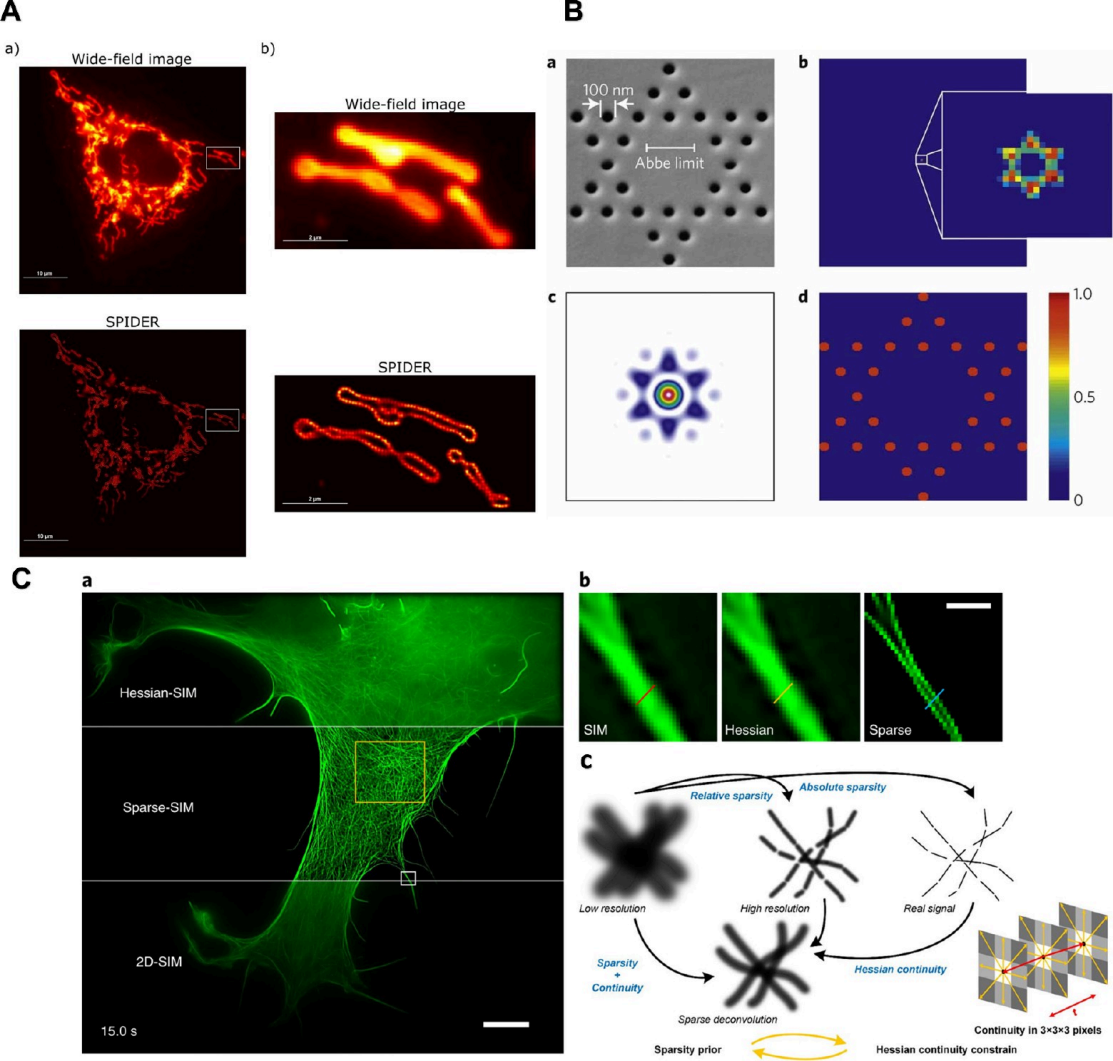
Results of three algorithms using the sparsity prior. (A)  minimization on Mitochondria imaging. Wide-field image: average live-cell image with local magnification. SPIDER:  minimization with a specific solve algorithm. Note that the membranes are recovered. (B)  minimization on coherent diffractive imaging. (a) A scanning electron microscope (SEM) image of the sample. (b) Blurred image, as seen in the microscope. The individual holes cannot be resolved. (c) The measured spatial power spectrum of the field. The color map is identical to that in the other panels: only the background has been removed for clarity. (d) The reconstructed 2D information algorithmically recovered from the measured power spectrum (c) and the blurred image (b). (C) Sparse-deconvolution on SIM achieves ∼60 nm resolution on the COS-7 cell labeled with (a) LifeAct–EGFP with (b) an enlarged region. (c) The concept of Sparse-deconvolution, see main text for details. (A) is reprinted by permission from ref (80). Licensed under a Creative Common Attribution (CC BY) 4.0 license. (B) is reprinted with permission from ref (82). Copyright 2012 Springer Nature. (C) Reprinted with permission from ref (10). Copyright 2021 The Authors.

The simplest sparsity is  norm sparsity. For a vector , if it contains only a small number of nonzero elements, that is,  norm of **x** is small, it is considered sparse. As shown in Figure 6(B(a)), the image (a 2D vector) has only a few significant points, making it a sparse image.

As mentioned, the CSR problem can be reformulated as an underdetermined equation-solving problem, written as
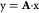
28where , with *m* < *n*. The sparsity prior assumes that the true solution is sparse. This type of problem is generally called a spare recovery problem. We refer to all solutions that satisfy the equation as feasible solutions. A reasonable approach is to find the sparsest solution among these feasible solutions. This approach can be formulated as a constraint optimization problem:
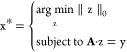
29

This is commonly referred to as  minimization. Suppose there is an algorithm that can solve this optimization problem , theoretical studies have shown that if the true solution is sufficiently sparse and the matrix **A** satisfies certain properties (see the mathematical definition in Supporting Information on  minimization), then the solution to  minimization is unique and exactly the true solution.^76,77^

Although this appears promising in theory, it misses a critical element: an efficient algorithm to solve  minimization. In fact, even before the theoretical research on uniqueness, by the 1990s it had been proven that  minimization is an NP-hard problem,^78^ meaning it cannot be solved in polynomial time. The only available methods involved exhaustive search or highly specialized algorithms, which limited its application to even moderately sized problems, let alone processing images in CSR.

This necessitated an alternative approach to  minimization. Before delving into that, it is worth noting that delineating the boundaries between tractable and intractable instances of  minimization remains an active research topic. In recent years, direct solutions to  minimization have seen renewed interest.^79^ For example, a 2016 study^80^ introduced a deconvolution algorithm for high-density emissive fluorophores in single-frame wide-field super-resolution imaging. This approach modeled high-density localization as  norm regularization and proposed an iterative algorithm called sparse image deconvolution and reconstruction (SPIDER) to solve  minimization. The study achieved SR imaging for mitochondria using total internal reflection fluorescence (TIRF) data (Figure 6(A)).

The difficulty of solving  minimization lies primarily in the properties of the  norm itself. In contrast, because the  norm is convex, its optimization is much easier:
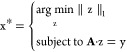
30In the literature, this is referred to as the basis pursuit (BP) problem. This raises a fundamental question: can  minimization replace  minimization? Using the concept of mutual coherence, it has been shown that if the true solution is sufficiently sparse and the matrix **A** satisfies certain properties, then the solution to  minimization is unique and corresponds to the true solution.^76,81^ Detailed definitions are provided in the Supporting Information on  minimization.

However, real-world signals are often not strictly sparse; they may not contain only a few nonzero elements. Instead, they are often “soft sparse”, meaning a small number of elements are large while many others are small but not exactly zero. This leads to the concept of compressibility.

In simple terms, a vector is considered compressible if setting most of its smaller elements to zero and retaining only the largest ones introduces only a small error compared to the original vector. Compressibility is closely related to sparsity, and vectors that are compressible but not strictly sparse are referred to as inexact sparse vectors.

Another source of inexactness is noise, which is not considered in the BP model. To address this, we introduce an error level η(e) corresponding to the real noise and modify BP into the following form:
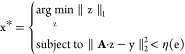
31

This optimization problem is named the quadratically constrained basis pursuit (QCBP). Notably, the minimizer of QCBP is not guaranteed to be unique.

The question then becomes, if the true signal is compressible, can QCBP recover or approximate the true image? Mathematically, is the error between any minimizer x* of QCBP and the true image bounded?

### Compressed Sensing

In 2006, milestone work by Donoho, Candès, Justin Romberg, and Terence Tao provided conclusive answers to these questions.^83,84^ Their contributions collectively became known as compressed sensing (CS) Theory, starting a revolution in signal processing.

The seminal works of Candès and Tao introduced the famous restricted isometry property (RIP).^85,86^ The detailed definition is presented in the Supporting Information on RIP. Briefly, if the true signal is compressible and matrix **A** satisfies the RIP (with a certain constant), then the error between any minimizer of QCBP and the true signal is bounded by a constant. This constant depends on factors such as sparsity, SNR, the RIP constant, and the compressibility error. When the true signal is sufficiently compressible and the SNR is high, this error becomes very small, meaning the solution recovered by QCBP is close to the true signal. Moreover, QCBP can be solved efficiently using existing algorithms.

With this, another problem remains: do matrices exist or can they be designed to satisfy the necessary properties for sparse recovery?

In practice, the ability to design matrix **A** is crucial, as the rows of **A** often have physical interpretations. For example, in magnetic resonance imaging (MRI), matrix **A** is a partial Fourier matrix, where each row represents a single MRI scan. Similarly, in other forms of imaging, the number of rows in **A** (denoted as *m*) corresponds to the number of samples taken. This is why **A** is referred to as the “sensing” matrix in CS. Traditionally, the minimal sampling required was constrained by the Shannon-Nyquist sampling theory.^87^

Thus, the refined version of the question becomes do matrices exist in practice that satisfy the requirements for sparse recovery, and what is the minimum number of samples required to guarantee this property?

Donoho, Candès, and Tao addressed this question using a special class of matrices: random matrices.^83,84^ Simply put, for three types of random matrices—randomly chosen rows from a unitary matrix, random Gaussian matrices, and random Bernoulli matrices—the required number of samples *m* can be much smaller than the dimension *n* while still satisfying RIP with high probability. Combined with earlier results, this means that if the true signal is sparse or compressible, then even when *m* ≪ *n*, QCBP can recover the true signal with high probability. This allows sparse signals to be reconstructed using far fewer samples than traditional methods require, which is the essence of the term “compressed” sensing. The mathematical definitions are provided in the Supporting Information on RIP Matrices.

With this, CS theory addresses a major question: it challenges the long-held Shannon–Nyquist sampling limit, revolutionizing the field of signal processing.

Naturally, CS has also impacted fluorescence imaging^88^ and super-resolution fields. In 2009, CS was first proposed as a method to recover sub-wavelength information from far-field optical images under coherent light imaging.^89^ The study assumed the object was spatially sparse and simulated low-pass filtering at different cutoff frequencies on the synthetic data. Using eq 30 for recovery, the results demonstrated SR, validating the feasibility of the method. Subsequently, the same team applied CS to partially incoherent light imaging,^90^ incoherent light,^91^ and coherent diffractive imaging^82^ (Figure 6(B)). In all cases, assuming sparsity, sparse recovery enabled SR to some extents.

From this point on, many subsequent works have directly applied CS to achieve SR, especially in cases like SMLM, where the data are genuinely sparse. For instance, the pioneering proposed CS-STORM, employing sparse recovery methods to reconstruct higher-density frames and reduce the number of frames needed for SMLM.^92^

On the other hand, the primary goal of CS itself is to reduce sampling rates. A more direct application involves designing optical devices to lower the number of elements or imaging instances required. A representative example is single-pixel imaging.^93^ In fluorescence microscopy, point-scanning techniques are often employed to encode sparsity, such as using CS to accelerate two-photon acquisition.^94^ These applications, yet, extend beyond the scope of CSR and can be explored in related references.^95^

However, a gap persists between the CS theory and CSR within the field of microscopy. The imaging matrix **P** in microscopy functions as a low-pass filter with deterministic sampling, which omits high frequencies and has not been demonstrated to meet the RIP. Indeed, to date, no deterministic matrix has been proven to satisfy RIP; all sensing matrices known to align with CS theory exhibit random elements. This issue has already been recognized within the CS field itself. In 2012, Candès remarked in another paper,^96^ “*This (super-resolution) is very different from a typical compressed sensing problem in which we wish to interpolate—and not extrapolate—the spectrum*.”

To address this issue, more advanced extensions of CS theory have been developed.^96^ Given the technical complexity of this topic, we provide a brief discussion in the Supporting Information.

### Generalized Sparsity Prior

As previously mentioned, sparsity refers to the knowledge that the true solution can be represented using only a few terms. In the context of the *l*_0_ norm, sparsity implies that the term is a single element of a vector. In this sense, both TV and DWT can also be considered forms of sparsity priors. Specifically, TV can be interpreted as a sparse transform in the gradient domain, where discontinuities in the image, such as edges, are sparse. Similarly, DWT achieves sparsity by representing an image in the wavelet domain. The combination of Compressed Sensing and DWT has been a significant success in MRI.^97^ Utilizing wavelet transformations to encode MRI images sparsely allows for reconstruction from undersampled data, greatly reducing the number of required scans and, consequently, saving both time and cost. This advancement has had significant historical importance in promoting CS.

The success of CS, particularly through the application of the  norm, has inspired the development of CSR methods that incorporate generalized forms of sparsity. These models are collectively referred to as low-dimensional sparse models.^98^ Given that this topic is beyond the scope of this Review and remains highly technical, with applications in biological imaging still under development, we will not delve into further details here. Interested readers are encouraged to consult the Supporting Information on “Generalized Sparsity Prior”.

### Optimization and Parameter Tuning

The introduction of nonlinear regularization terms brings new challenges to optimization. For example, TV and  minimization involve nonsmooth functions that cannot be directly addressed using gradient descent. This necessitates the development of specialized optimization algorithms. Another challenge is parameter tuning, particularly the adjustment of λ in the objective function. Given the technical complexity of these topics, interested readers are encouraged to refer to the Supporting Information on “Operator Splitting Optimization Methods and Parameter Tuning”.

### Sparse-Deconvolution

One major advancement in model-based CSR is sparse-deconvolution, proposed by Zhao et al. in 2022.^10^ This method marks a breakthrough in general-purpose CSR.

As mentioned earlier, RL deconvolution has inherent super-resolution capabilities; however, its performance degrades in the presence of noise. Sparse-deconvolution leverages a priori knowledge about the sparsity and continuity of biological structures to enhance the resolution through deconvolution algorithms. This principle is illustrated in Figure 6(C).

The continuity prior assesses the relationship between pixel values along both spatial and temporal axes. According to sampling theory, the FWHM of the PSF is at least two pixels in the data, making sparse pointillist structures continuous within that region spatially. Additionally, if the movement of the biological sample between two consecutive time points is smaller than the lateral resolution, then the structures are considered continuous along the temporal axis. In contrast, noise typically lacks continuity along these axes. The continuity prior to this is quantified using the Hessian matrix continuity, which regularizes second-order gradients. This approach is derived from Hessian–Schatten regularization^99,100^ but employs the  norm instead:
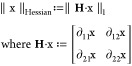
32where ∂_*pq*_x represents the second-order differential operator along image directions *p* and *q*.

Compared to TV, the Hessian prior represents a significant advancement in reducing nonimage features. It indeed shows that the “*Hessian-based regularizer is most effective for describing locally smooth features present in biological specimens*”.^101^ Prior to the advent of sparse-deconvolution, Hessian regularization was combined with Wiener-SIM to propose Hessian-SIM.^9^ This approach achieves artifact-minimized super-resolution (SR) images using less than 10% of the photon dose required by conventional SIM, while substantially outperforming current algorithms at low signal intensities.

Another important prior is sparsity, but it differs from  norm sparsity. An increase in spatial resolution in any fluorescence microscope always results in a smaller PSF. Compared with conventional microscopy, the convolution of the object with this smaller PSF in SR imaging leads to a relative increase in sparsity. This sparsity prior is modeled using the  norm. (Note that this background is different from compressed sensing.)

Additionally, sparse deconvolution accounts for the removal of background light by decomposing the image using wavelets and retaining only the first few coefficients. This approach has proven to be a crucial factor in the success of the algorithm.

The basic function of sparse-deconvolution is
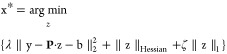
33where *b* is background light to remove and λ and ξ are regularization parameters.

Different from iterative deconvolution, sparse-deconvolution does not solve the optimization function directly but iteratively solves it in two steps:
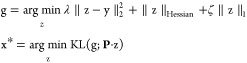
34The second step corresponds to RL. Experiments show that the best super-resolution effect can be achieved only by two-stage optimization.

Sparse-deconvolution improves resolution and contrast compared to raw RL. The resolving power of sparse-deconvolution was verified by comparing the results of known structures (nuclear pores) with SMIM. Combined with SIM, sparse-deconvolution achieves ultrafast 60 nm resolution in live cells (Figure 6(C)). Sparse-deconvolution has universal live-cell super-resolution and improved resolution of spinning-disc confocal SIM (SD-SIM), wide-field, confocal, two-photon, and expansion microscopes.

Subsequent research has explored one-stage optimization approaches, but it consistently demonstrates that combining sparsity and continuity priors is the most effective method.^102^ However, the impact of sparse-deconvolution also raises additional questions, such as its theoretical foundation and parameter adjustment, among other concerns.

### Note on Model-based CSR

Model-based CSR represents substantial progress compared with earlier explorations. However, several significant challenges remain. First, while model-based CSR employs handcrafted priors that are effective, these priors lack a unified design approach and may not generalize well across diverse applications. Second, stronger theoretical foundations are needed to justify the use of these priors and their outcomes. Additionally, parameter tuning and ensuring computational feasibility remain difficult challenges. Notably, there is no effective method for solving nonconvex regularization.

Despite these hurdles, model-based CSR has laid a strong foundation for the field. While it is still too early to draw definitive conclusions about model-based CSR, we can refer to the famous quote: “*All models are wrong, but some are useful*.”

## Data-Driven CSR

While model-based CSR relies on manually designed priors, an entirely different approach avoids explicitly modeling these priors and instead uses genuine high-resolution images as examples. By leveraging the features of these images to constrain the super-resolution results, the data itself serve as the prior; these are known as “data priors”.

To implement this approach, tools capable of directly extracting features from the data are essential. In computer science, machine learning is one such tool. This shift has been accelerated by the rise of deep learning^22^ (DL), a specific machine learning method that relies on data to optimize artificial neural networks (ANNs) to approximate complex function mappings. DL has revolutionized various fields, including CSR.

**Figure 7:**
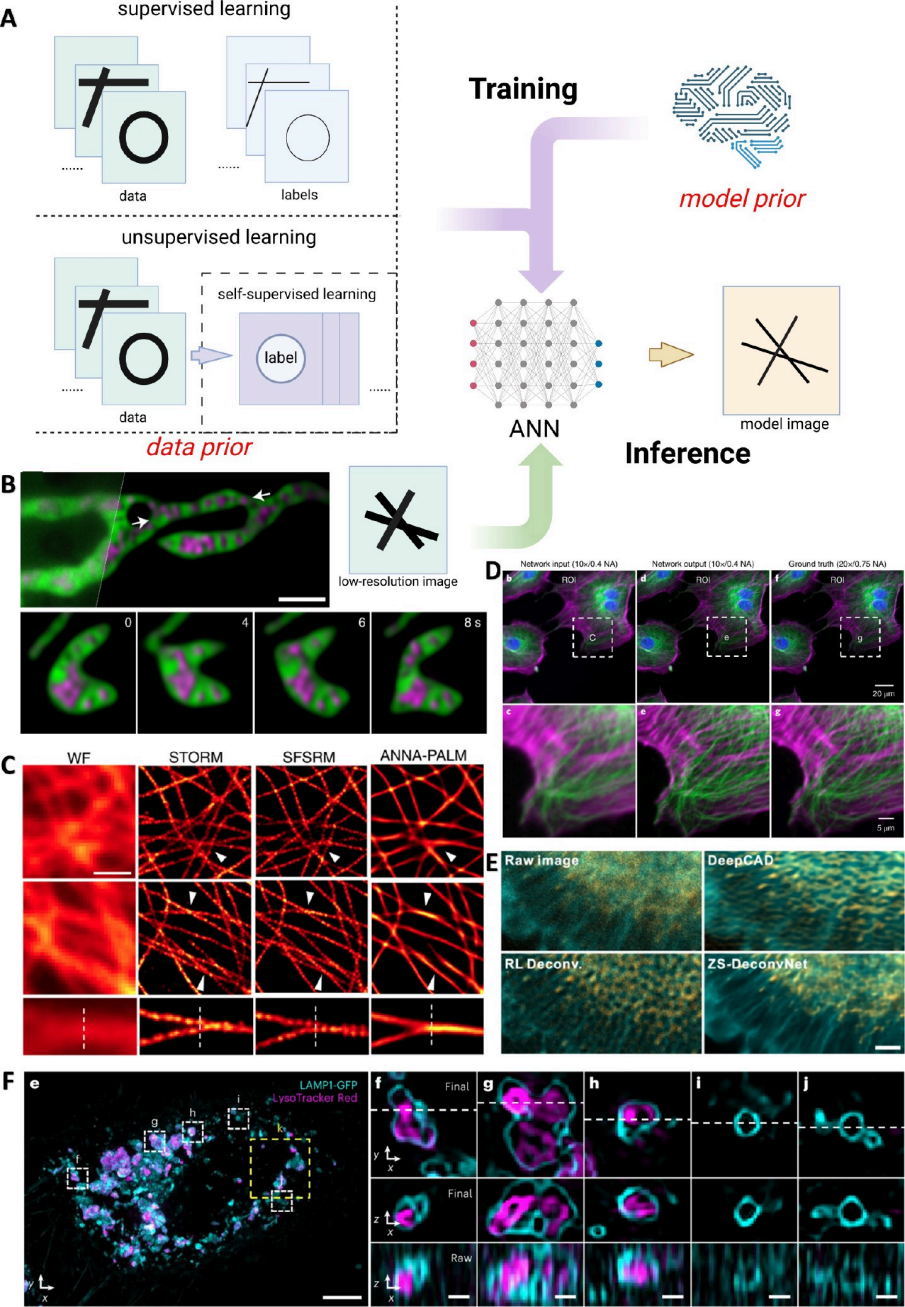
Concepts and representative works in DL-CSR. (A) Workflow of deep learning for CSR. Details are provided in the main text. (B–D) Supervised learning CSR. (B) Representative two-color DFCAN images of the mitochondrial inner membrane (green) and nucleoids (magenta). Top left: a fraction of the corresponding wide-field image. Scale bar, 2 μm. (C) SFSRM’s performance on immunostained microtubules in fixed Beas2B cells. WF: wide-field; ANNAPALM is a deep learning model for SMLM reconstruction.^108^ Scale bar: 1 μm. (D) Super-resolved images of bovine pulmonary artery endothelial cells (BPAECs) by a GAN SR model. (E) Unsupervised learning CSR. Representative SR images reconstructed by ZS-DeconvNet of the F-actin cytoskeleton (cyan) and myosin-II (orange). DeepCAD is a self-supervised denoising model.^109^ Scale bar: 5 μm. (F) Self-supervised CSR for 4D SR with isotropic resolution. Results of the cascaded deep learning process on live U2OS cells expressing lysosomal marker LAMP1-GFP (cyan) and additionally labeled with LysoTracker Red to mark the lysosome interior (magenta). Scale bars: 5 μm (left large subfigure) and 500 nm (right small subfigures). (A) was created in https://BioRender.com. (B) is reprinted with permission from ref (110). Copyright 2021 The Authors. (C) is reprinted with permission from ref (111). Copyright 2023 The Authors, licensed under a Creative Common Attribution (CC BY) 4.0 license. (D) is reprinted with permission from ref (11). Copyright 2019 The Authors. (E) is reprinted with permission from ref (112). Copyright 2024 The Authors, licensed under a Creative Common Attribution (CC BY) 4.0 license. (F) is reprinted with permission from ref (113). Copyright 2023 The Authors, licensed under a Creative Common Attribution (CC BY) 4.0 license.

This section introduces deep learning for CSR (DL-CSR). For readers unfamiliar with DL, we briefly explain its foundational concepts (Figure 7(A)), although a comprehensive understanding will require consulting more detailed tutorials.^103^

### Deep-Learning

Suppose we have a large data set of paired low-resolution (LR, e.g., wide-field microscopy) and high-resolution (HR, e.g., super-resolution fluorescence microscopy) images. Denote the LR images as {*y*_*i*_}_*i* = 1_^*N*^ (termed *data*) and their corresponding HR images as {*x*_*i*_}_*i* = 1_^*N*^ (termed *labels* or *ground truths*). The imaging process that connects them can be approximated by *y*_*i*_ ≈ psf⊗*x*_*i*_, where the approximation accounts for noise.

Supervised machine learning refers to fitting a model using these paired data to transform *y*_*i*_ to *x*_*i*_. Typically, the model is parametrized, and these parameters are learned by minimizing the average value of a loss function that quantifies discrepancies between the predicted HR output and the true HR label. This process is called training. If the training process uses a data set without labels but only data, it is called unsupervised machine learning. A special unsupervised learning is self-supervised learning; it generates pseudo-labels from data only and trains the model as supervised learning. After training, we expect the trained model to infer accurate HR outputs for new LR inputs outside the training set, a phase known as inference. The ability of the model to perform well on unseen data that were not part of the training set is called generalization.

A major challenge in machine learning is the risk of overfitting.^114^ Overfitting occurs when a model memorizes the training data too well but fails to generalize to unseen data. To determine whether the model can generalize and to guide training, a validation data set is needed. Additionally, to address overfitting, alongside increasing the amount of training data, regularization techniques are required, a concept familiar in CSR.

DL is a type of machine learning in which the model is an artificial neural network (ANN). Inspired by the structure of neural connectivity in the brain, we designed an ANN consisting of multiple layers of artificial neurons. Each artificial neuron, known as a node, maps inputs to outputs through a mathematical function:
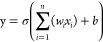
35where *x*_*i*_ represents a single input variable (among *n* such inputs), *w*_*i*_ represents a weight for that input, *b* represents a learnable bias term, and σ represents a nonlinear activation function that takes a single input and returns a single output. The weights and biases are the parameters to be fitted in an artificial neuron. Collectively, the parameters of all artificial neurons are the parameters to be fitted in an entire ANN.

A key property of ANNs is that a two-layer ANN is sufficient to approximate almost any (with a slight request) mathematical function to an arbitrary level of accuracy, provided there are enough artificial neurons. This capability may explain the success of DL. In a simple ANN, the output of one layer serves as the input to the next (Figure 7(A)). Alternatively, there are various approaches for arranging artificial neurons in more complex structures. For image applications, a popular architecture is a convolutional neural network (CNN).

Supervised deep learning uses data and corresponding labels to train an ANN. The primary tool for this process is stochastic optimization. In each iteration, the ANN randomly selects a small batch of the data set, uses the data in the batch as input, and computes the output. The error between the output and labels is back-propagated to each node in the network in a process called backpropagation. The ANN then uses gradient descent to update the parameters based on the calculated error. After a sufficient number of iterations, the ANN reduces the loss function on the training set to a sufficiently small value.

If the ANN learns all transformations automatically during training, mapping raw data directly to the desired output, the framework is called end-to-end. Yet, it proves to be suboptimal when a particular prior occurs for specific task, such as in CSR. Thus, useful model priors can be designed to enhance DL performance and sometimes even essential for generalization.^104^

Once training is complete, the ANN can be deployed to generate new low-resolution images for SR. DL-CSR can also be integrated into the broader framework of the basic function:
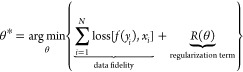
36where *f* represents the ANN and θ are its parameters.

The main flow of the DL is outlined above. Due to its powerful feature extraction capabilities, these principles are fundamentally the same across different domains. In fact, the primary difference between DL-CSR in microscopy and DL natural image SR lies in the data used to train the DL models. Therefore, readers interested in the broader scope of DL-based SR can directly refer to reviews on natural image SR.^105−107^ Here, we focus specifically on representative advancements in DL-CSR for microscopy.

### Supervised Learning CSR

Variations in supervised learning CSR methods typically arise from three main aspects: the training data, the neural network architecture, and the specific model priors used.

First, the quality and nature of the training data significantly influence the performance of the DL model. The most straightforward approach is to collect paired data using bimodal methods. For example, one of the earliest works in DL-CSR utilized two data sets: one used different numerical apertures (10×/0.4-NA and 20×/0.75-NA) in wide-field microscopy, and another used confocal and STED imaging to generate paired LR and HR images.^11^ In some cases, specific protocols are required to generate matched data; for instance, Xu et al. performed in vitro imaging of acute slices of SEP-GluA2 brains to produce a paired data set, aiming to improve the resolution of in vivo two-photon imaging.^114^

However, obtaining paired data is very challenging, especially when imaging with different modalities, where registration becomes difficult. Additionally, maintaining the consistency of the sample before and after imaging is hard. When physical methods for generating paired data are impractical, computational approaches can be employed. One strategy is to collect high-resolution images and simulate the corresponding low-resolution counterparts. For example, Qiao et al. used SIM to acquire high-resolution images of four cellular structures under varying illumination conditions.^110^ They averaged these SIM images to generate wide-field images at different SNR levels, thus creating paired data sets. Similarly, Wang et al. first acquired high-resolution point-scanning images and then used a wave optics model of light-field microscopy to project these high-resolution 3D images into 2D light-field images for training.^115^ Despite this progress, acquiring high-resolution images is still time-consuming and resource-intensive, especially when SR fluorescence microscopy is required.

Another significant aspect is the evolution of neural network architectures. Using CNNs as basic building blocks, various complex structures can be constructed. Among these, the most commonly used architectures for image processing tasks are U-Net^116^ and 3D-Unet,^117^ which follow an encoder–decoder architecture. CARE (content-aware image restoration) was one of the earliest works to apply U-Net to microscopy image restoration^118^ and has since become the benchmark for many subsequent DL-CSR models. For example, XTC (cross-trained CARE) utilized the CARE structure for SR tasks using previously mentioned data from ex vivo super-resolution and in vivo imaging modalities.^114^ Another structural advancement involves the use of attention mechanisms,^119^ which dynamically adjust the weights between artificial neurons, allowing the network to learn more representative features. A representative work in computer vision that employs this technique is RCAN (residual channel attention networks).^120^ Later, it was used in SR microscopy to denoise and extend resolution^121^ and was extended to DFCAN (deep Fourier channel attention network) incorporating attention mechanisms in the frequency domain.^110^

U-Net has also been integrated into generative adversarial networks (GANs).^122^ By combining a generator and a discriminator, GAN-based models enhance the network’s capabilities. For instance, Wang et al. used a GAN model to transform diffraction-limited input images into super-resolved ones.^11^ Chen et al. proposed single-frame super-resolution microscopy (SFSRM), further employed enhanced super-resolution generative adversarial networks (ESRGAN),^123^ and introduced improved loss functions to enhance the reliability of CSR training results.^111^ In addition to these advancements, network architectures also vary in the design of loss functions, specific network layers, and other implementation details; for details, see more comprehensive reviews.^105−107^

The third major development is task prior utilization, modeled as a regularization term. Since SR is inherently an ill-posed problem, deep learning models are particularly prone to overfitting. Introducing explicit regularization can improve the model generalizability. Beyond the commonly used regularization methods in DL,^124^ incorporating domain-specific priors has been shown to be highly effective.^104^ These priors can be embedded into the network structure or the loss function. A prime example is DFCAN, which uses a Fourier channel attention (FCA) mechanism to exploit the characteristics of the power spectrum of distinct feature maps in the Fourier domain, resulting in more stable training and improved generalization. Additionally, Chen et al. used edge maps from low-resolution images as guidance to design a multicomponent regularizer.^111^ Bouchard et al. proposed TA-GAN (task-assisted generative adversarial network), which employs auxiliary tasks, such as segmentation or localization, to guide SR training.^125^ These results demonstrate that while DL-based methods are inherently data-driven, introducing priors is a critical step in stabilizing networks and improving generalizability, similar to model-based CSR.

### Unsupervised Learning CSR

The lack of sufficient paired data is a major bottleneck in broadly applying supervised learning to CSR. In the context of live-cell imaging, obtaining SR images as ground truths is often infeasible. Unsupervised learning provides a potential solution to this challenge but introduces additional difficulties. Currently, applications of unsupervised learning in CSR are much less common than supervised approaches and are not yet developed enough to form a conceptual category; we can discuss only existing case studies.

One typical scenario stems from the high cost of acquiring high-resolution images, while it is generally easier to obtain abundant low-resolution data. Qiao et al. proposed ZS-DeconvNet (zero-shot deconvolution networks), where the objective function is given as^112^
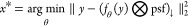
37Here, θ represents the neural network parameters, *f*_θ_ denotes the network, and ↓ refers to downsampling. Zero-shot implies that high-resolution data are not used to train *f*_θ_. However, directly training with this objective can lead to instability. To address this, ZS-DeconvNet employs an additional denoisingr network and a Hessian regularization term to stabilize convergence. The denoisingr network, named R2R, is trained in a self-supervised manner, which will be introduced in the following paragraph. ZS-DeconvNet enhances the resolution of microscope images by more than 1.5-fold over the diffraction limit, with 10-fold lower fluorescence than ordinary super-resolution imaging conditions. Nevertheless, even though it is labeled as zero-shot, training the neural network still requires a large amount of data, and generalization confers still a problem.

Self-supervised learning is a special case of unsupervised learning, where pseudolabels are generated from unlabeled data and used to train neural networks in a supervised fashion. In the R2R (recorrupted-to-recorrupted) example above, a single noisy image is transformed to generate two new noisy versions, which are then treated as data-label pairs for network training.^126^ Under certain assumptions, R2R is theoretically proven to be equivalent to supervised learning. The principle behind self-supervised learning is that supervision signals are inherently present in the data and can be extracted by specific methods.

A notable application of self-supervised learning in CSR is improving the axial resolution. Axial resolution in microscopy is typically much lower than the lateral resolution. By treating lateral 2D slices from 3D data sets as high-resolution images and simulating low-resolution images computationally, neural networks can be trained and later applied to infer axial 2D images. For example, Li et al. combined this approach with optical setup adjustments to enhance SIM to achieve ∼120 nm isotropic resolution.^113^ Park et al.^127^ further employed cycleGAN to eliminate the need for computationally generated low-resolution images, training directly on unpaired lateral and axial 2D images.^128^ Although unsupervised and self-supervised CSR research remains limited, these approaches hold promise for future advancements.

### Note on Deep-Leaning CSR

DL-CSR employs a data-driven framework, extracting priors directly from training data unlike model-based CSR, which relies on handcrafted prior modeling. Given abundant data, an appropriate network architecture, and well-tuned training processes, DL-CSR often surpasses traditional methods. Moreover, once trained, DL-CSR models are both fast and flexible, making them suitable for specific tasks.

We present the results of several representative studies mentioned above in Figure 7(B–F). As shown for supervised learning examples, the SR images generated by DFCAN from wide-field mitochondrial images can clearly resolve mitochondrial inner membranes and nucleoids^110^ (Figure 7 (B)), SFSRM achieves super-resolution of wide-field images to match the results of SMLM^111^ (Figure 7 (C)), and cross-modality SR digitally enhances low numerical aperture (10×/0.4-NA) images to high numerical aperture quality^11^ (Figure 7 (D)). Figure 7(E) illustrates unsupervised CSR, showcasing long-term SR imaging by using ZS-DeconvNet. It achieves super-resolution of the F-actin cytoskeleton and myosin-II, which are dynamic and photosensitive biological processes.^112^Figure 7(F) demonstrates the application of self-supervised learning CSR to achieve isotropic 4D SIM resolution.^113^

However, the comparison between model-based CSR and DL-CSR is nuanced. DL-CSR’s performance is highly dependent on the quality and size of the training data. Obtaining large, high-quality, diverse data sets for CSR is challenging, particularly in live-cell imaging scenarios requiring ultrafast and ultrahigh-resolution imaging. In such cases, SR ground truth data are often entirely unavailable, rendering DL-CSR incapable of learning the SR mapping. While unsupervised learning in CSR offers some promise, progress in this area is still limited. Conversely, model-based CSR can be applied under these challenging conditions.

Another significant challenge is generalization. Training data for DL-CSR often include only a limited set of cellular structures. When faced with the task of super-resolving novel structures, the performance of DL-CSR can degrade significantly. In contrast, model-based CSR typically exhibits much better generalization capabilities.

Stability is another growing concern for DL methods, particularly in CSR. For instance, experiments have shown that DL models for MRI reconstruction can produce unstable outputs—minor changes in the test image can lead to drastic differences in the reconstruction, or vice versa.^129^ This instability could result in severe clinical consequences, such as misidentifying tumors or overlooking critical pathological features. As DL models become increasingly complex, instability issues may worsen, leading to hallucinations, a topic we will explore further in the next section on Foundation models.

A more fundamental issue is that DL-CSR remains a black box to this day, which is particularly challenging for the field of SR, where interpretability is paramount.^130^ We concur with the view^131^ that “*if based on a solid mathematical model of the imaging and noise processes, carefully executed deconvolution should be the preferred choice for post-processing techniques*”.

Despite these challenges, several promising trends are emerging to address these issues. The field of DL itself is progressing toward solutions, as researchers across various disciplines work on adopting DL responsibly. Research on interpretable DL aims to demystify the “black box” nature of these models.^132^ Until significant breakthroughs are achieved, a practical approach involves providing uncertainty metrics to inform users of potential risks, with Bayesian DL offering a viable pathway.^133^

For CSR, the key challenge is data dependency, highlighting the need for more high-quality, publicly available data sets; data sets like BioSR serve as an inspiring example.^110^ Additionally, further applications of unsupervised learning can help mitigate some of these data-related constraints.

Finally, considering the complementary strengths and weaknesses of model-based and data-driven approaches, a promising direction is their integration. Combining these methodologies could leverage their respective advantages, leading to more robust and effective CSR solutions. This leads us to the latest advancements in CSR, which will be introduced in the next section.

## The Future of Convergence of Models and Data

Since the 2010s, there has been a growing advocacy for a “fourth paradigm”^134^ of research driven by data-based scientific discovery.^135^ The debate between models and data is not new. A historical example is the contrast between fitting planetary orbits using detailed observational data and Newton’s derivation of laws. In the context of CSR, we, like many across various fields, believe that these approaches are not mutually exclusive.^136^ Rather, their integration and complementary advancement present the most promising path forward.

Emerging as the cutting-edge trend in CSR is the convergence of model-based and data-driven methods. We will draw on recent works from the past several years to trace this trend and illustrate how combining these methodologies can lead to significant progress.

### Image Pre-Processing

Fluorescence imaging faces fundamental limitations due to various degrading factors, particularly stochastic shot-noise inherent in photon detection, which restricts the quality of images recorded by cameras. CSR is especially sensitive to noise; as the analytical continuation theorem suggests, deconvolution can theoretically achieve infinite resolution under noise-free conditions. However, in live-cell imaging, photon budgets are often restricted, necessitating the use of moderate or low light intensities, which increase noise levels.

Recent advancements in DL image preprocessing have led to transformative improvements.^137^ Sophisticated self-supervised denoising methods enable high SNR imaging with significantly fewer photons and in some cases even surpass the shot-noise limit.^138^ This has made the combination of advanced DL denoising techniques and CSR algorithms a highly promising trend.

One work by Qiao et al. proposed a rationalized deep learning (rDL) approach.^139^ By leveraging the physical properties of SIM patterns to constrain network outputs, this method significantly enhances the denoising of raw SIM images. This results in over 10-fold improved SR reconstruction at lower illumination intensities compared to other computational approaches. Another notable example introduced a novel DL-based denoising method using specific fluorescence label methods with reduced data dependency.^140^ This method is combined with sparse-deconvolution to achieve single-frame live-cell super-resolution imaging, demonstrating the potential of integrating advanced DL techniques with CSR methodologies.

In addition to noise, background light—comprising defocused signal light and scattered light—significantly affects imaging quality. Effective background removal is crucial for sparse-deconvolution to achieve robust CSR. However, methods like wavelet-based background suppression often improve the resolution at the expense of removing weak signals or altering reconstruction linearity. A notable example is BF-SIM, which introduced a physical model-based background suppression algorithm optimized for 2D-SIM. This approach demonstrated that effective background removal can enhance the fidelity of sparse-deconvolution results.^141^

Other factors, such as defocusing and fluorescence drift, also impact the image quality. Continued advancements in DL-based low-level image processing are anticipated to yield increasingly higher-quality images. Therefore, obtaining the highest-quality images before applying CSR algorithms is crucial to achieving optimal results.

### Foundation Models

Recently, two novel neural network architectures, namely, transformers^142^ and the diffusion model,^143^ have driven significant progress in DL, supported by large data sets and specialized training methods. This progress has culminated in the emergence of Foundation Models.

Originally developed for natural language processing, transformers employ complex attention mechanisms. When combined with massive data sets, transformers enable a new training paradigm called pretraining. This approach relies on unsupervised techniques, especially self-supervised learning, to extract intrinsic data structures from large data sets prior to fine-tuning for specific downstream tasks. Due to their enormous parameter sizes and remarkable generalization capabilities, these models are referred to as foundation models,^144^ with large language models (LLMs) being a prime example. Transformers have since been adapted for imaging tasks, leading to the development of image foundation models.^145^

In microscopy, traditional supervised learning methods are often limited to specific tasks such as CSR, requiring task-specific data sets for optimal performance. Ma et al. applied foundation model methods to microscopy restoration,^146^ leveraging the Swin transformer (an improved version of the transformer).^147^ They developed a foundation model called UniFMIR, which was trained on various restoration tasks using an aggregated large data set comprising 196 418 training samples from 14 public data sets (approximately 30 GB). UniFMIR demonstrated a strong performance on downstream single-image SR tasks, effectively reconstructing details in wide-field images to achieve SIM-level resolution. This success was evident across diverse cellular structures, including clathrin-coated pits, endoplasmic reticulum, microtubules, and actin filaments.

Another architecture, the diffusion model, is based on highly dimensional sampling processes and is particularly well-suited for image data. Beyond serving as discriminative foundation models, diffusion models support generative learning,^148^ an unsupervised approach that learns the probability distribution of data from large unlabeled data sets. This capability enables the generation of new, unseen data.

For supervised CSR, where obtaining high-resolution images can be challenging, generative learning offers a potential solution by creating new data sets from limited existing high-resolution data. Saguy et al. provided a proof-of-concept in bioimaging by training a diffusion model on SR fluorescence data of several organelles.^149^ The generated data were then used to train new supervised models for single-image SR, yielding results comparable to those from traditional data collection approaches.

However, generative approaches remain contentious in scientific research due to the risk of hallucinations, a common issue found in foundation models.

#### Hallucination of the Foundation Model

Foundation models have introduced a new challenge: hallucination.^150^ In practice, these models may generate outputs that are misaligned with their training data or actual phenomena. For example, large language models sometimes produce a nonsensical text. This phenomenon, which is akin to DL instability, currently lacks a comprehensive explanation. In CSR, this uncertainty limits the broader adoption of foundation models as it becomes difficult to discern whether the results represent genuine SR details or artifacts.

Despite these challenges, foundation models have already made a significant impact globally; just consider the widespread recognition of ChatGPT.^151^ Their potential in CSR remains promising, and ongoing research may address these limitations to fully leverage their capabilities.

### Hybrid of Model and DL Methods

Combining the strengths of physical priors and data-driven DL capabilities has emerged as a key trend in CSR. Here are some notable recent approaches:

#### Deep Image Prior (DIP)

Traditionally, the power of DL is attributed to priors derived from training data. Contrary to this belief, DIP demonstrates that the architecture of an untrained neural network itself can capture significant low-level image statistics, functioning as a form of regularization.^152^ In CSR, DIP replaces traditional regularization terms with an untrained neural network:
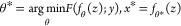
38Here, *z* is a fixed random noise vector and *f*_θ_ represents the network structure. The minimizer θ* is obtained using an optimizer such as gradient descent starting from a random initialization of parameters θ. The result of the high-resolution image is obtained as x* = *f*_θ*_(*z*). Without any training data, DIP showed remarkable SR performance. Incorporating model-based regularization (e.g., TV) further enhances results, highlighting the potential of DIP to mitigate DL-CSR’s dependency on data.

#### Learned Prior

Learned priors use DL to design regularization terms in the basic function. An instance is total deep variation (TDV).^153^ TDV is a convolutional neural network that extracts local features on multiple scales and in successive blocks. TDV is set as regularization term in the basic function, and optimization is performed in optimal control formulation. The integrated TDV and SIM reconstruction in ref^154^ , named TDV-SIM, outperforms conventional or DL methods in suppressing artifacts and hallucinations while maintaining resolutions.

#### Plug-and-Play Method

Plug-and-play (PnP) methods^160^ integrate DL-based denoisiers as proximal operators in model-based CSR optimization, enhancing stability while retaining the original CSR structure. Though widely used in general image restoration, their application in microscopy-specific CSR remains underexplored.

#### Algorithm Unrolling

Algorithm unrolling replaces iterative optimization steps in traditional methods with neural network layers.^161^ This approach automates parameter tuning using data, improving stability and inference speed. For example, Li et al. developed the Richardson–Lucy network (RLN), a 3D microscopy deconvolution method that combines the forward/backward projector structure of RL deconvolution with DL.^156^ Benchmarks show that RLN produces fewer artifacts compared to RL or purely data-driven models while requiring fewer parameters.

These examples illustrate the trend of integrating model-based and data-driven frameworks. For a more comprehensive overview, readers can refer to ref (136).

### Miscellaneous Specialized Methods

Emerging CSR methods also leverage unique physical priors. For example, SUPPOSE (super position of virtual point sources) is similar to optimization-based deconvolution but models the SR target as a collection of virtual points of identical intensity.^157^ This approach results in a nonconvex optimization problem, which is originally solved using genetic algorithms. Subsequently, Jiang et al. replaced genetic algorithms with gradient descent to develop A-PoD (adaptive moment estimation optimization-based pointillism deconvolution).^13^ A-PoD demonstrates a superior SR performance in Raman imaging. Another major class of CSR algorithms departs from deconvolution-based structures, utilizing fluorescence fluctuation information for SR. SRRF (super-resolution radial fluctuations) achieve SR imaging by analyzing radial gradient variations in fluorescence fluctuation signals,^164^ while eSRRF redefines key principles for estimating radiality and temporal analysis to enhance image reconstruction quality.^165^ By optimizing parameter ranges and acquisition configurations, eSRRF minimizes artifacts and nonlinear distortions, improving the overall fidelity to the underlying structure. MSSR (mean-shift super resolution) is a combination of SRRF and single-molecule deconvolution.^158^ MSSR enhances image resolution by iteratively shifting pixel intensities toward the local mean within a defined neighborhood, achieving approximately 2-fold resolution improvement.

Inspired by fluorescence fluctuations, an image-sharpening approach based on pixel reassignment was introduced to general microscope modalities and various fluorophore types.^159^ The main steps involve first preconditioning the raw image and then performing pixel reassignment, where pixel values are reassigned to neighboring locations according to the direction and magnitude of the localized image gradient.

## Applications

In this section, we show the applications of CSR in biological imaging, particularly in advancing live-cell SR imaging and enabling biological discoveries.

### Breaking the Photon Budget limit

Live-cell SR imaging poses significant challenges as it requires a delicate balance within a certain photon budget. CSR effectively addresses these barriers by extracting super-resolved information computationally while retaining the advantages of lower-resolution imaging methods, such as higher acquisition speed, improved SNR, and reduced phototoxicity.

For example, traditional deconvolution algorithms enhance the photon utilization efficiency of conventional microscopy. By incorporating advanced priors, such as continuation and sparsity constraints, sparse-deconvolution on SIM achieves approximately 60 nm resolution at frame rates up to 564 Hz.^10^ This capability allows researchers to resolve intricate structures, including small vesicular fusion pores, ring-shaped nuclear pores formed by nucleoporins, and the relative movements of inner and outer mitochondrial membranes in live cells.

Thanks to these advantages, CSR has been successfully applied to studies of mitochondrial nucleoid dynamics within mitochondrial cristae,^110^ rotational streaming of mitochondrial tubes, mitochondrial fission, cell division,^121^ and cytoskeletal dynamics in immune cells,^113^ as well as subcellular growth cone dynamics in living *C. elegans* embryos.^166^ The inferred high-resolution images provide more accurate segmentation and reveal finer details than those available from lower-resolution raw data, driving new insights into dynamic cellular processes.

### Facilitating Biological Discoveries

CSR has powered live-cell SR imaging, enabling new observations and insights. Here, we illustrate its transformative impact through four examples spanning subcellular structures (actin), functional proteins (nuclear pore complexes), neural cells, and preclinical disease studies.

#### Actin

**Figure 8:**
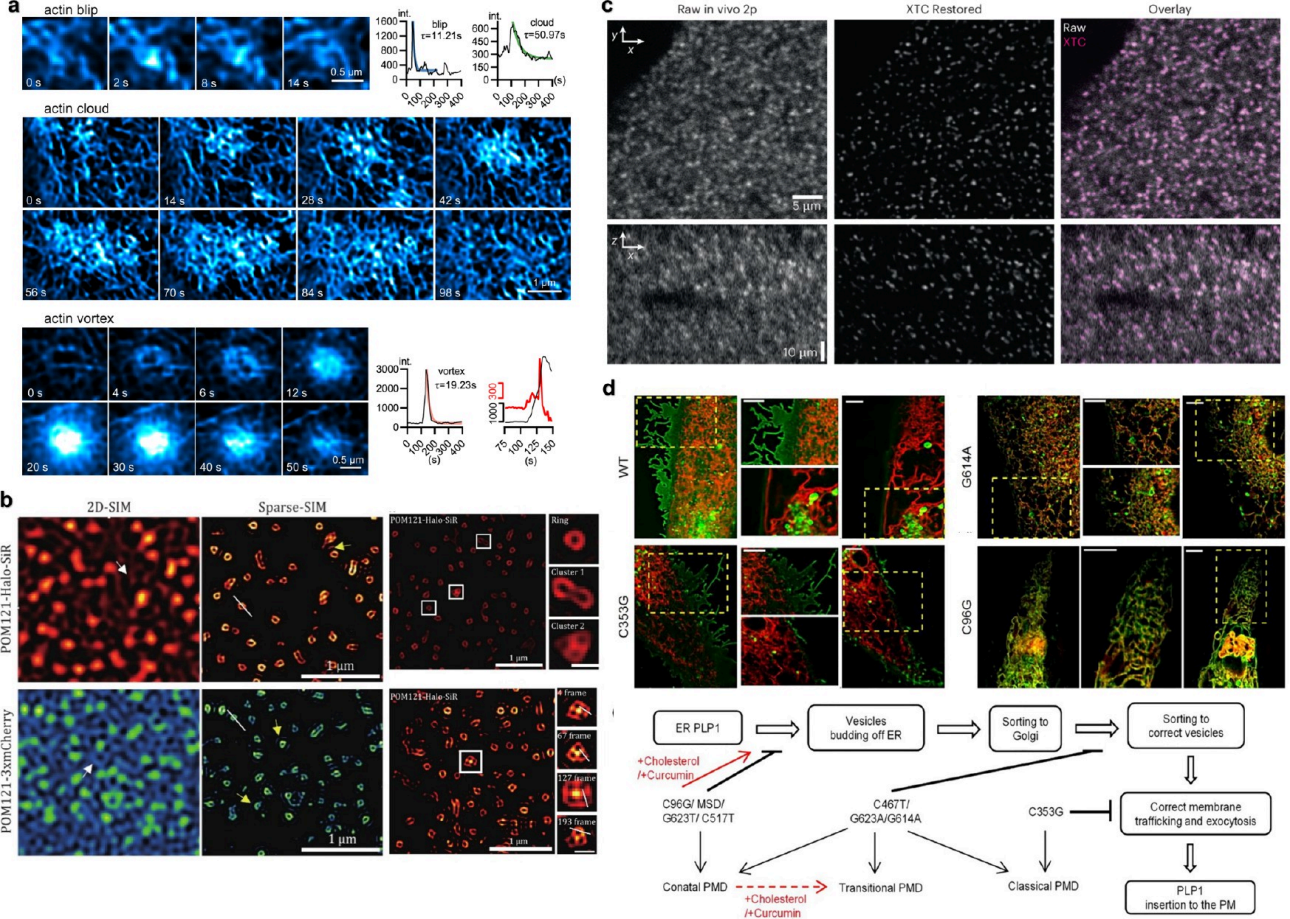
Biological discoveries enabled by CSR. (a) Three types of localized transient actin dynamics: an actin blip event that was small in size, an actin cloud event that was large, and an actin vortex event that was medium in size. (b) A representative example of nuclear pores labeled with POM121-Halo or POM121-3xmCherry in a live MCF7 cell. Two-dimensional structured illumination microscopy (2D-SIM) was reconstructed with the Wiener algorithm (first column), and sparse-SIM was reconstructed with the sparse-deconvolution algorithm (second column). NPC morphology and morphological dynamics (third column). (Top) A representative example of different nuclear pore structures labeled by POM121-Halo. (Bottom) A snapshot of the petaled gathering nuclear pore structure enclosed by a white box. The white box on the right is enlarged and shown at four time points to show the dynamic changes of nuclear pores. (c) XTC super-resolves SEP synapses in vivo. Comparison of the same in vivo 2p image before (left) and after XTC (middle). All images show a single axial slice. (d) Live-cell super-resolution pathology enables precise identification of genotype–phenotype correlations with different PLP1 mutations. The images show that MO3.13 cells were cotransfected with different proteins (green) and the ER marker protein Sec 61β (red). The scheme shows defects at different steps of the trafficking of PLP1 mutants to the plasma membrane in different PMD subtypes and the function of cholesterol or curcumin in facilitating the escape of the severe PLP1 mutants from ER. (a) is reprinted by permission from ref (141). Copyright 2023 The Authors, licensed under a Creative Common Attribution (CC BY) 4.0 license. (b) is reproduced with permission from ref (162). Licensed under a Creative Common Attribution (CC BY) 4.0 license. (c) is reprinted with permission from ref (114). Copyright 2023 The Authors, licensed under a Creative Common Attribution (CC BY) 4.0 license. (d) is reprinted with permission from ref (163). Copyright Science China Press, licensed under a Creative Common Attribution (CC BY) 4.0 license.

Actin forms gel-like, dynamic networks under the cell cortex with pore diameters of 50–200 nm. Resolving these networks requires both high spatial and temporal resolution, which is challenging for conventional live-cell SR methods. Using Sparse-SIM,^10^ researchers achieved sub-70 nm resolution and visualized weakly labeled actin filaments in live macrophages. Enhanced with advanced background subtraction,^141^ this technique facilitated the discovery of transient actin dynamics, including “actin blips” (localized puncta that emerge and disassemble), “actin clouds” (collaborative assemblies of actin spreading laterally), and “actin vortexes” (ring-like structures with spiral brightening) (Figure 8 (a)). These confirm CSR’s ability to reveal intricate cytoskeletal behaviors critical for cellular function.

#### Nuclear Pore Complexes (NPCs)

NPCs are enormous, 8-fold symmetrical protein assemblies. The limited number of fluorophores labeling these proteins, which correlates with their few copy numbers, sets an upper limit on the photon budget for conventional SR fluorescence microscopy. Using labeled NPC proteins with Halo-SiR fluorophores and employing Sparse-SIM, 20 Hz imaging with enhanced contrast and photostability is achieved.^162^ This method resolved fine NPC structures and uncovered atypical morphological changes in the NPC clusters (Figure 8(b)). These findings demonstrate CSR’s capability to study dynamic protein assemblies in live cells at extreme spatiotemporal resolutions.

#### Synaptic Plasticity

Learning involves changes in glutamate receptors at synapses, which mediate neuronal communication. However, the sub-micrometer size and high density of synapses make them difficult to resolve in vivo, limiting the study of receptor dynamics during behavior. To address this challenge, a transgenic mouse line (SEP-GluA2) was developed, along with a computational pipeline that combines a DL image-restoration algorithm (XTC) with in vivo imaging^114^ (Figure 8(c)). This approach enabled super-resolution tracking of AMPA receptor (AMPAR) dynamics at synapses during behavior. By linking spatiotemporal changes in AMPAR content to synaptic strength and behavior, CSR has provided new insights into the molecular underpinnings of learning and memory.

#### Pelizaeus–Merzbacher Disease (PMD)

CSR has also made significant contributions to clinical research, as demonstrated by its application to Pelizaeus–Merzbacher disease (PMD), a hypomyelination leukodystrophy disorder.^163^ Using sparse-SIM, researchers investigated PLP1 mutations associated with PMD, identifying three distinct cellular phenotypes linked to clinical subtypes (Figure 8(d)): severe phenotypes resulting from ER retention, intermediate phenotypes involving lysosomal missorting, and mild phenotypes related to vesicle trafficking defects. This study provided the first cellular-level characterization of PMD subtypes, uncovering their pathogenic mechanisms and paving the way for targeted therapeutic strategies.

In summary, the transformative applications of CSR across diverse biological contexts demonstrate its immense potential to revolutionize biological research.

### Integrating CSR with Multimodal Imaging

CSR can be seamlessly integrated with various imaging modalities. Below, we highlight CSR’s applications in several advanced imaging techniques, including SOFI, Raman microscopy, spatial omics, and expansion microscopy, showcasing its transformative impact on biological research.

#### SOFI

**Figure 9:**
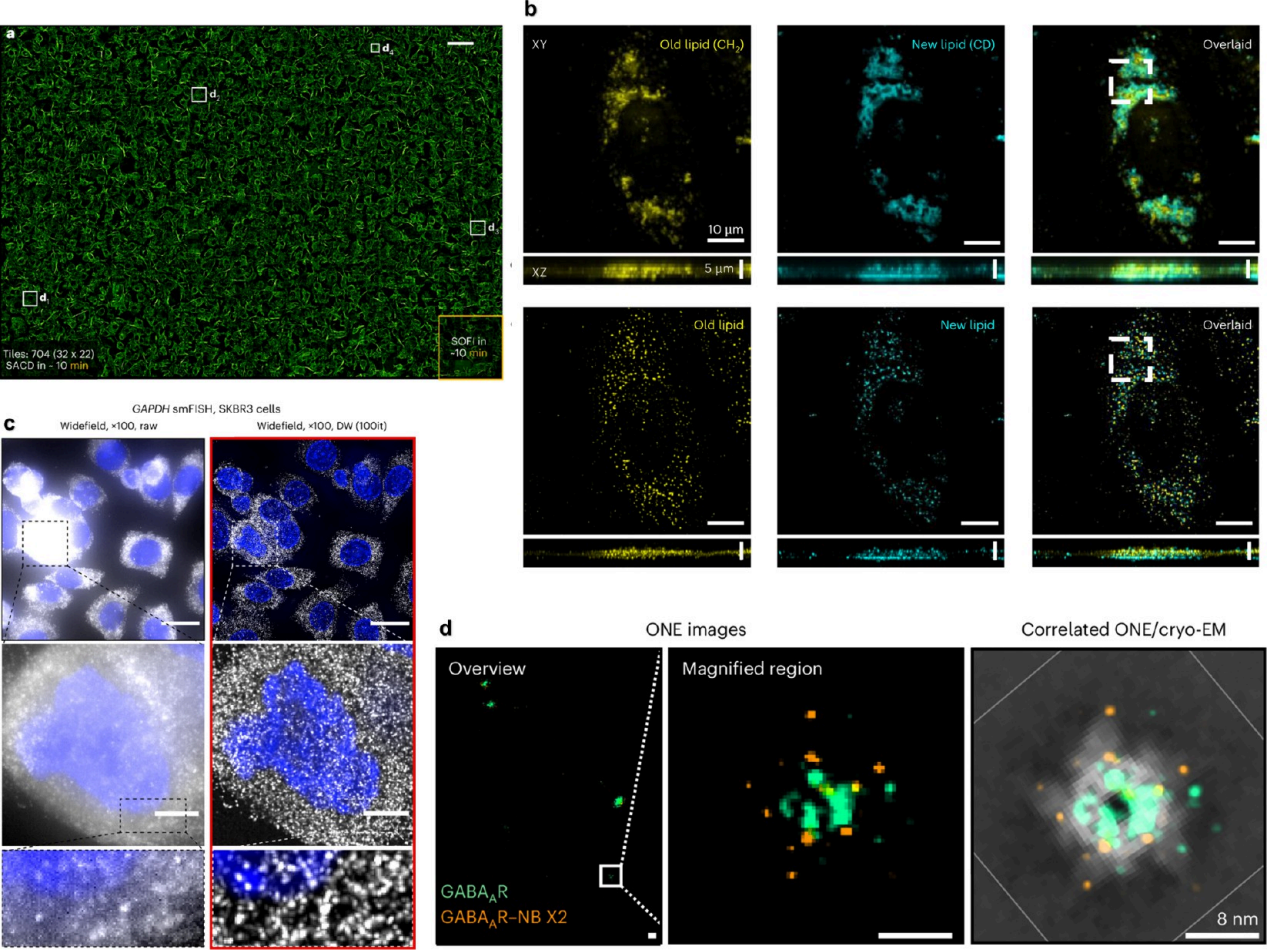
Integration of CSR with multimodal imaging. (a) Application of SACD (with 20 frames) to high-throughput SR imaging of an ∼2.0 mm × 1.4 mm area containing more than 2000 cells. Microtubules are identified in COS-7 cells labeled with QD605. Scale bar, 0.1 mm. (b) (Top) DO-SRS images of LDs in CH2 and CD channels. The CH2 channel represents the distribution of old LDs (left), and the CD vibration image shows the distribution of newly synthesized LDs (middle). To compare the two images (left and middle), the images were overlaid (right). (Bottom) DO-SRS images were deconvolved using A-PoD, and the results clearly separate the signals of two different types of LDs, old versus newly synthesized (left, middle, and right) (c) Human SKBR3 cells stained with smFISH probes targeting GAPDH transcripts (white) (left) and after deconvolution with DW (right). Imaging: widefield, ×100 oil objective (NA 1.45). Maximum z-projection is shown. Blue, DNA. Scale bars, 20 μm in top panel, 5 μm in middle panel. (d) Superimposition of ONE microscopy images and cryo-EM data. The overview panel shows an exemplary ONE image (from a total of 648 ONE images, acquired from at least six gels) of GABAAR–Nb that are postexpansion labeled with NHS-ester dyes, followed by a magnified region of a single receptor. The last panel shows a cryo-EM–ONE overlay. [(a) is reproduced with permission from ref (12). Copyright 2023 The authors. (b) is reproduced with permission from ref (13)). Copyright 2023 The Authors. (c) is reproduced with permission from ref (14). Copyright 2024 The Authors, licensed under a Creative Common Attribution (CC BY) 4.0 license. (d) is reproduced with permission from ref (167). Copyright 2024 The Authors, licensed under a Creative Common Attribution (CC BY) 4.0 license.

CSR methods have been employed to improve SOFI by addressing noise and background artifacts. For example, the SACD (autocorrelation with two-step deconvolution) technique introduces RL deconvolution to preprocess raw image sequences before calculating autocorrelated cumulants.^12^ This preprocessing step effectively removes out-of-focus and cytoplasmic background noise, enhances the effective switching contrast, and reduces random pixel-level noise while maintaining linearity across frames. By incorporating Fourier interpolation and advanced postprocessing, SACD achieves 2-fold improvements in lateral and axial resolution with as few as 20 raw image frames (Figure 9(a)). This integration exemplifies how CSR can significantly enhance the spatiotemporal resolution of the SOFI.

#### Raman Microscopy

Stimulated Raman scattering (SRS) microscopy enables imaging of metabolic dynamics with high signal-to-noise ratios but is spatially constrained by the numerical aperture of the objective and the scattering cross-section of molecules. CSR addresses these limitations through algorithms such as the A-PoD.^13^ A-PoD achieved a spatial resolution below 59 nm on lipid droplet membranes, enabling a detailed analysis of protein and lipid distributions within cells. Further, A-PoD-enhanced deuterium oxide-probed SRS (DO-SRS) imaging differentiated newly synthesized lipids in lipid droplets and revealed metabolic changes in *Drosophila* brain samples under different diets (Figure 9(b)). This integration demonstrates CSR’s capability to enhance vibrational spectroscopy, offering nanoscopic insights into biomolecular dynamics and spatial localization.

#### Spatial Omics

CSR has proven to be particularly impactful in microscopy-based spatial transcriptomics, where densely packed molecular targets often exceed the diffraction limit. For instance, Deconwolf, an open-sourced accelerated version of the RL algorithm,^14^ improves the identification of transcripts in wide-field fluorescence microscopy. Applied to DNA and RNA fluorescence in situ hybridization (FISH) images, Deconwolf enhances transcript detection more than 3-fold and facilitates chromosome tracing with fluorescence in situ sequencing of barcoded probes (Figure 9(c)). By enabling robust quantification of diffraction-limited fluorescence dots, CSR expands the utility of spatial omics techniques, offering a detailed molecular view of biological systems.

#### Expansion Microscopy

CSR also enhances expansion microscopy (ExM) by further improving resolution and SNR. For instance, when SRRF is combined with ExM, it achieves near-molecular-scale precision (Figure 9(d)).^167^ Using an isotropically expandable X10 gel and acquiring thousands of frames, SRRF analyzes radial symmetries and higher-order temporal statistics to reconstruct high-resolution images. By mitigating imaging drift and optimizing the SNR, this approach can separate fluorophores found at distances of approximately 20 nm, achieving resolutions close to 10 nm in ideal conditions. This combination of CSR and ExM allows for detailed visualization of biological structures, with significant implications for studying complex molecular assemblies.

These advancements unlock new possibilities for studying dynamic biological processes, revealing insights into molecular, cellular, and tissue-level structures with unparalleled precision.

## Artifacts and Resolution Quantification in CSR

In scientific research, the primary goal of SR imaging is fidelity. With the rapid development of SR technologies, objectively measuring the resolution achieved by SR methods has become a critical challenge in the field.^131^

Systematically, the uncertainty (or error) of resolution can arise from two main sources: “model bias” and “data bias”. Model bias results from discrepancies between the estimation model and the physical reality that it aims to represent. In CSR, the mismatch between prior models and the actual biological structures is an inherent limitation that affects the SR results. Data bias primarily stems from noise, mechanical inaccuracies of the imaging system, and other random factors. Both model bias and data bias can introduce artifacts, undermining the fidelity of the SR imaging.

Traditionally, analytical formulations have been used to analyze microscopy resolution by modeling factors such as system aberrations and noise. Yet, for CSR, relying solely on theoretical analysis to quantify resolution is often impractical. This challenge is further exacerbated in DL-CSR, where the underlying models are more opaque.

The most objective approach to validate resolution and detect artifacts is to compare CSR results against a ground-truth (GT) reference. Calibration samples such as DNA origami structures^168^ or known biological structures like nuclear pore complexes^169^ can serve this purpose. For biological samples, higher-resolution techniques such as electron microscopy can sometimes be used as a reference.

However, in most biological and chemical studies, GT is often unavailable due to either technical limitations or the nature of the experiment (e.g., live-cell imaging).

A classical method for assessing data bias without GT is Fourier ring correlation (FRC),^170^ which has been adapted for the SR domain:^171^
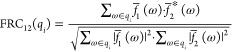
39

The FRC method measures the statistical correlation between two signals over a series of concentric rings in the Fourier domain. Here, *f*_1_ and *f*_2_ are statistically independent images of the object by like imaging the identical object with the same configurations. ∑_ωϵ*q_i_*_ represents the summation over the pixels on the perimeter of circles of the corresponding spatial frequency *q*_*i*_.

The bias of the data can be highlighted by the difference between *f*_1_ and *f*_2_. However, the “absolute differences” is susceptible to the intensity fluctuation and subpixel structural motions, so classical FRC has difficult detecting the local heterogeneity of resolution.

To address this, rolling FRC (rFRC)^172^ was proposed. This approach applies FRC locally using a rolling block, akin to a moving filter, with thresholds chosen for specific spatial frequencies. Validation in different SR imaging shows the effectiveness and stability of rFRC.

While FRC and rFRC are effective for detecting data bias, they are not feasible for identifying model bias, which is often embedded in SR reconstructions. A notable method for addressing this issue is SQUIRREL (super-resolution quantitative image rating and reporting of error locations).^173^ SQUIRREL evaluates SR errors by comparing them to a simultaneously acquired high-SNR wide-field reference.

rFRC can further be combined with SQUIRREL using a rolling block map to create a composite map known as PANEL (pixel-level analysis of error locations), which pinpoints regions with low reliability for subsequent biological profiling.

Nevertheless, detecting the model bias remains extremely challenging without a GT reference. For instance, there has been a long-standing debate in SR regarding whether the honeycomb artifacts observed in SIM arise from system design or background light.^174,175^ When fidelity cannot be directly assessed, reproducibility becomes the next best measure of SR reliability. CSR has an advantage here: compared to experimental environments that are prone to uncontrollable variability, the physical randomness in the CSR is minimal. By thoroughly documenting all parameters and intermediate processes, reproducibility can be ensured—except for methods involving stochastic elements such as certain DL-based CSR approaches.

On the other hand, CSR, particularly model-based methods, offers an advantage in dealing with artifacts through its foundation in mathematical principles. These methods allow researchers to detect and reduce patterned artifacts systematically, compensating for gaps in the biological knowledge. For example, systematic patterns in textures can often be traced back to their computational origins and corrected.

Finally, even if certain phenomena observed in SR images cannot be fully validated, CSR still has a unique advantage: low cost and nondestructive processing. Unlike experimental methods that may consume samples or require costly resources, CSR preserves the original image data while providing valuable insights. This makes CSR an excellent “compass” for guiding exploratory biological research. Researchers can use CSR to identify potential areas of interest before committing to more complex and expensive techniques.

## Challenges and Outlook of CSR

After reviewing more than half a century of development in CSR, its future appears to be promising. Of course, challenges remain. This section outlines the current major challenges and prospects for future advancements.

### Ultra-High Resolution Ultra-Fast Live-Cell Imaging

Ultrahigh-resolution and ultrafast live-cell imaging remains a critical frontier in the field of CSR. Achieving this ambitious goal requires the ability to visualize dynamic cellular processes in real-time with nanometer-scale resolution while preserving cell viability and minimizing phototoxicity. Although significant progress has been made, CSR is still far from fully realizing this objective, which presents both key challenges and exciting opportunities.

First, achieving ultrahigh-resolution and ultrafast imaging demands the development of more advanced algorithms, which in turn requires foundational theoretical research. Key areas include the design of innovative priors, theoretical guarantees for reconstruction accuracy, improved model interpretability, and algorithmic stability under challenging conditions.

The emergence of foundation models presents another promising avenue. With sufficiently large data sets and computational resources, could CSR reach a singularity point where the problem is fundamentally solved? Testing this hypothesis requires the construction of large biomedical imaging models, which necessitates overcoming challenges, such as data sharing, multimodal data integration, and large-scale data processing.

Second, CSR must evolve alongside the latest advancements in optical microscopy. For example, MINFLUX (MINimal photon FLUXes), a breakthrough in fluorescence microscopy, achieves nanometer-scale resolution and has enabled live tracking of motor proteins.^176^ However, it faces challenges such as low throughput and limited temporal resolution. Integrating CSR with MINFLUX’s localization algorithms could reduce the number of images required, enhance SNR, and increase imaging speed.

Additionally, CSR can be paired with light-sheet microscopy to minimize photobleaching by selectively illuminating only the imaging plane. This integration could enable high-resolution imaging with reduced phototoxicity, preserving the sample integrity during long-term observations. Another exciting prospect lies in expanding CSR-compatible imaging modalities to include label-free methods.^177^ Techniques such as phase imaging or label-free scattering hold promise for minimally invasive live-cell imaging. By reducing the reliance on fluorescent labels, these methods could enable CSR to be applied in scenarios in which fluorescence imaging is impractical or detrimental to the sample.

### Quantitative CSR

The ultimate goal of improving resolution is to enable quantitative analysis of biological processes on finer scales. The fundamental requirements for quantitative live-cell CSR are preserving the integrity of delicate structures and maintaining the linearity of fluorescence signals. Although this area largely remains uncharted territory, model-based CSR grounded in accurate mathematical and physical models holds significant promise.

Moreover, CSR algorithms should aim to facilitate enhanced quantitative processing and the segmentation of biological phenomena. In this context, DL-based detection and segmentation methods have already demonstrated remarkable capabilities. Integrating advanced CSR algorithms with DL for quantitative analysis is an essential step toward making CSR a universal tool for life sciences.

### Unknown or Spatially Variant PSF

Most methods discussed in this Review rely on the assumption in imaging (eq 1) that the PSF is precisely known and spatially invariant. However, this assumption often presents challenges in practice.^178^ Aberrations and scattering can make it difficult to accurately obtain the PSF, and local PSF variations may arise—especially in advanced microscopy techniques like light-sheet or light-field microscopy, where the PSF is inherently spatially variant.

Addressing unknown or spatially variant PSFs in CSR remains a difficult problem. Blind deconvolution^179^ offers a potential solution by simultaneously estimating the PSF and performing deconvolution, though this significantly increases the complexity. DL may bypass this issue by learning PSF information directly from data; however, acquiring sufficient ground-truth data remains infeasible. Adaptive optics^180^ can correct the PSF to reduce uncertainties. Recently, advancements in deconvolution theory for a spatially varied PSF^181^ have opened new avenues of exploration.

### Computational Imaging

Historically, CSR and fluorescence SR microscopy have developed largely independently. One notable exception is SIM, which encodes high-frequency signals into the imaging system and uses CSR algorithms to recover them. Recently, similar approaches—combining algorithm and hardware design—have gained increasing attention under the umbrella of computational imaging.^182^

Computational imaging integrates computation as a fundamental part of the image-formation process. CSR leverages priors, and the introduction of more suitable priors through system design can foster a symbiotic relationship between algorithms and hardware, potentially enabling the next generation of SR imaging systems. A successful precedent is single-pixel imaging, which incorporates CS theory into a system design. The development of more controllable hardware systems has accelerated this trend. For instance, metamaterials allow for precise tailoring of light–matter interactions at subwavelength scales, opening up possibilities for designing more controllable and reliable systems in conjunction with CSR algorithms.^183^

### Orientation to Practical Applications

Every biotechnique should ultimately aim for practical applicability. Perhaps the greatest challenge for CSR lies in how to make it accessible to end-users (e.g., biologists and chemists) for their research. Like SR fluorescence microscopy, CSR faces high adoption barriers due to its complexity. To address this, CSR researchers must engage in an interdisciplinary collaboration with biologists and chemists. Additionally, designing more user-friendly hardware and software systems is imperative.

Beyond the integration of more reliable algorithmic tools, a promising trend is leveraging foundation language models to create intuitive user interfaces. Such interfaces could allow users to operate CSR-based smart microscopy^184^ systems without requiring in-depth understanding of the underlying principles, significantly lowering the barrier to entry.

## Summary

In the pursuit of breaking imaging barriers, the CSR represents a testament to the boundless ingenuity of science. This Review has traced the odyssey of CSR. Starting with fluorescence microscopy as a model, we introduced the process of optical imaging. Using Fourier transform as a tool, we derived the frequency-domain definition of resolution and, subsequently, the meaning of super-resolution. CSR achieves super-resolution through algorithms, with its core relying on the introduction of priors to solve ill-posed problems. We have unified diverse CSR methods under a single conceptual framework, defined by the rule of priors and the interplay of computation. Within this framework, we have traced the historical development of CSR, beginning with its roots in analytical continuation and early iterative deconvolution, moving through paradigm-shifting advancements in model-based and data-driven methods and culminating in recent breakthroughs such as sparse-deconvolution and deep-learning-based CSR. Along the way, we highlighted the biological applications of CSR in overcoming photon budget limitations and extending the boundaries of live-cell super-resolution imaging. Besides, we discussed the objective resolution quantification method.

Yet, as much as CSR has advanced, its journey is far from complete. The challenges of achieving ultrahigh resolution and ultrafast live-cell imaging require more advanced theories and algorithms. Additionally, integrating interdisciplinary knowledge, designing interpretable models, and overcoming barriers to adoption among biologists remind us that CSR is not just a computational endeavor but a dynamic collaboration between physics, biology, and computation.

Alongside state-of-the-art super-resolution fluorescence microscopy, CSR promises to be integrated into the next generation of SR imaging systems. This integration is poised to deliver unprecedented precision and detail with the potential to revolutionize biological research.

Swiss-born American naturalist, geologist Louis Agassiz (1807–1873) once wrote a poem that thought-provokingly revealed the evolution of scientific breakthroughs, which is displayed in the entrance area of the Nobel Museum in Stockholm:^185^

“Every great scientific truth goes through three stages. First, people say it conflicts with the Bible. Next they say it had been discovered before. Lastly they say they always believed it.”

Even in the era of artificial intelligence, CSR stands as a profound reminder that it is not algorithms or data but our ideas, wisdom, curiosity, and boundless courage to challenge the limits of nature that shape the future.

## Supporting Information Available

All mathematical concepts and proofs that are covered in the main text but not specifically elaborated (PDF)
