# Supplementary material for: Computational Super-Resolution: An Odyssey in Harnessing Priors to Enhance Optical Microscopy Resolution
Source: Anal Chem. 2025 Feb 27;97(9):4763–92. doi: 10.1021/acs.analchem.4c07047 (PMC11912138; doi:10.1021/acs.analchem.4c07047)
Supplement: Supplementary file 1 — ac4c07047_si_001.pdf [file ac4c07047_si_001.pdf]

Supporting Information of Computational Super-Resolution: An Odyssey in Harnessing Priors to Enhance Optical Microscopy Resolution

Wenfeng Tian, Riwang Chen, Liangyi Chen*

Computational Super-Resolution (CSR) and Nature Image Super-Resolution. In natural image SR, high-resolution (HR) means that pixel density within an image is high. The aim of nature image SR is to increase pixel resolution from observed multiple low-resolution (LR) images, also known as *digital super-resolution*[1], *geometrical super-resolution*[2], pioneering by Tsai and Huang[3]. As the standard book said, "(Natural image) Super-Resolution (SR) are techniques that construct high-resolution (HR) images from several observed low-resolution (LR) images…The non-redundant information contained in the these LR images is typically introduced by subpixel shifts between them"[4]. **In this sense, natural image SR is an upsampling challenge, where the extracted information can include both high-frequency and low-frequency components. This differs from CSR, which aims to recapture only the high-frequency information.** Nevertheless, some digital SR methods can be applied in microscopy, particularly in time-lapse microscopy imaging.

A closely related technique to natural image SR is the single-image interpolation approach, also known as *image zooming* or *magnification*, which can also be used to increase image size. However, this method does not provide additional high-frequency information. What really deserves attention in computer vision is single-image super-resolution (SISR), a concept closely related to CSR.

The goal of SISR is to infer information on a finer grid based on data available on a coarse grid from a single LR image. SISR developed relatively late, and until the beginning of the 21st century, it was considered impossible to achieve[1] (which explains why traditional natural image SR refers to multi-images SR). SISR shares a key characteristic with CSR: both problems are ill-posed and require prior information and specialized algorithms for inference. Consequently, many approaches overlap between the two fields. The advent of DL has further blurred the lines between CSR and SISR; many DL-based methods originally developed for SISR have been adapted for CSR in microscopy.

Despite the methodological similarities, CSR and SISR differ in several aspects. CSR models often include precise (theoretical) representations of optical and noise effects, whereas SISR typically relies on simplified imaging assumptions, often modeling the forward process as basic downsampling. This has led to the challenge known as the real-world SR problem[5]. Another subtle difference is that SISR deals with undersampled images, while CSR handles usually oversampled images. Therefore, SISR operates at the pixel level, which does not necessarily correspond to out-of-band extrapolation, while CSR operates at the frequency domain level.

Details of Imaging Process.

PSF. For incoherent imaging (such as fluorescence microscopy), if the exit pupil is circular with a radius of a, focal length is f_0, the PSF for a laser of wavelength λ is given (after normalization) by:

$$\mathrm{PSF}(r) = \mathrm{jinc}^2\left(\frac{2\pi a r}{\lambda f_0}\right)$$
$$\text{where } \mathrm{jinc}(x) = \frac{J_1(x)}{x} \tag{1}$$

where J_1 is the *first-order Bessel function*. The formula for the OTF is too complex to include here; interested readers can refer to Chapter 4 in Ref.[6].

Sampling. While the underlying sample is continuous, both the input and output are represented in discrete form during the imaging process. This naturally leads to considerations regarding sampling. The classical sampling theorem states that sampling must occur at a rate at least twice the cutoff frequency to avoid aliasing[7]. For a low-resolution image y this condition is typically satisfied in optical microscopy[8]. For a high-resolution image x, the sampling rate represents the SR objective we aim to achieve.

3D OTF. The OTF is a three-dimensional frequency domain function, as depicted above. The OTF exhibits a central depression in its vertical cross-section, known as the "missing cone," where the OTF values are zero. Physically, this implies that conventional optical microscopes cannot distinguish between light originating from different axial positions. Biological imaging samples are generally three-dimensional, meaning regions outside the focal plane also emit fluorescence, referred to as "out-of-focus" or "background light." Since the OTF cannot differentiate between focal plane and background light, conventional microscopes lack *optical sectioning* capability, which leads to reduced image contrast. Consequently, the effective resolution is even lower than the theoretical limit imposed by the cutoff frequency.

Thus, SR in microscopy involves not only extending the frequency spectrum but also addressing the missing cone problem—essentially improving optical sectioning capability.

Convolution as Matrix Multiplication. For the convolution equation, first, the two-dimensional image x (similarly for three-dimensional images) is rearranged row by row into a column vector $\mathbf{x}$. Next, the PSF is constructed as a matrix $\mathbf{P}$. Each row of $\mathbf{P}$ corresponds to a row vector derived from the raw PSF, with each successive row shifted one position to the right relative to the previous row using periodic boundary conditions. This construction makes $\mathbf{P}$ a *Block Circulant with Circulant Blocks* (BCCB) matrix[9]. The BCCB matrix can be decomposed by the Fourier matrix:

$$\mathbf{P} = \mathbf{F}^{\mathrm{T}} \cdot \mathbf{O} \cdot \mathbf{F} \tag{2}$$

Where $\mathbf{O}$ is OTF matrix. It is a diagonal matrix whose diagonal elements are rearranged from the OTF vector. Due to the low-pass filtering property of the OTF, the diagonal elements of $\mathbf{O}$ are zero at both ends. As a result, the BCCB matrix is not of full rank. The rank of the BCCB matrix is the number of non-zero elements in the OTF vector.

Convex and Non-Convex. Convexity is a property of a function. A function is convex if, for any two points within its domain, the straight line connecting those two points lies entirely above or on the graph of the function, and not bellowing it. For example, the ℓ_1 norm function is convex. In contrast, a function is non-convex if the straight line between two points can lie below the function's graph in some places. For example, the ℓ_0 norm function is non-convex.

Convexity is important in optimization because it ensures that any local minimum is also a global minimum. This means that optimization algorithms, such as gradient descent, can reliably find the best solution without getting stuck in local minima.

In non-convex problems, there may be many local minima, making it harder to find the global minimum, and the optimization process becomes more complex and uncertain.

Lagrange Multiplier Method. The Lagrange multiplier method is a mathematical optimization technique used to find the maximum or minimum of a function subject to constraints. It transforms the constrained optimization problem into an unconstrained optimization problem.

For example, the constraint optimization problem:

$$x^* = \begin{cases} \arg\min_x f(x) \\ \text{subject to } g(x) \le 0 \end{cases} \quad (3)$$

The *Lagranian* is

$$L(x,\lambda) = f(x) + \lambda g(x) \quad (4)$$

$\lambda \ge 0$ is Lagrange multiplier.

With Lagranian, define the *primal problem* is

$$x^* = \arg\min_x L(x,\lambda) \quad (5)$$

and the *dual problem* is:

$$\lambda^* = \arg\max_{\lambda \ge 0} \inf_x L(x,\lambda) \quad (6)$$

under certain conditions[10], the optimal (x^*, λ^*) is the solution of primal and dual problems, and x^* is the solution to constraint optimization problem. Thus, we can solve the primal unconstrained optimization problem directly. Although this method introduces another variable, it can make the constrained optimization problem easier to solve under certain conditions.

Bayesian Views of Priors. Another perspective on priors in CSR comes from Bayesian statistics, which models the prior as a probabilistic constraint[11]. Methods based on this approach are often referred to as *statistical model methods*. In this framework, all variables, such as the true high-resolution image and noise, are treated as random variables following specific probability distributions. The prior $p(x)$ represents the probability distribution that characterizes all possible true high-resolution images.

Under Bayesian equation, *the posterior distribution* $p(x|y)$, given the observed data y, can be expressed as:

$$p(x \mid y) = \frac{p(y \mid x)p(x)}{p(y)} \quad (7)$$

Here, $p(y|x)$ is the likelihood, which depends on the forward imaging process and the noise distribution.

We look for the solution that best matches the posterior probability. One approach is the *Maximum a Posteriori* (MAP) solution. Since $p(y)$ is observed and fixed, maximizing the posterior distribution is equivalent to:

$$x^* = \arg\max_z p(z \mid y) = \arg\max_z p(y \mid z)p(z)$$

(8)

Taking the negative logarithm (a monotonic transformation) simplifies the problem to:

$$x^* = \arg\min_z \{-\log p(y \mid z) - \log p(z)\} \quad (9)$$

In practice, the prior distribution $p(x)$ is often modeled as a Gibbs distribution[12]$(\frac{1}{Z}e^{-\lambda r(x)})$. Here, $r(x)$ is a penalty function representing the prior, λ is a regularization parameter, and Z is a normalizing constant that ensures $p(x)$ is a valid probability distribution. Substituting this into the MAP framework yields:

$$x^* = \arg\min_z \{-\log P(y \mid z) + \lambda r(z)\} \quad (10)$$

This MAP formulation closely resembles the basic framework discussed earlier, where $-\log p(y|z)$ corresponds to the data fidelity term and $r(z)$ corresponds to the regularization term.

Furthermore, modeling the posterior probability has additional advantages. Rather than simply finding the MAP estimate, measuring the entire posterior distribution provides more comprehensive information about the CSR. For instance, it can offer insights into the likelihood of obtaining a true image and quantify the associated uncertainty. However, this approach comes with a trade-off: computing the full posterior distribution is more challenging and often requires computationally expensive sampling techniques or approximations, such as Markov Chain Monte Carlo (MCMC) or variational inference. For more advanced treatments of Bayesian inverse problems, please refer to this paper[13].

A class of statistical model is *Markov random field* (MRF). It is based on MAP framework

Equation (10). The MRF assumes that an image pixel is only influenced by the pixels in its surrounding neighborhood, and thus models the prior distribution as:

$$\frac{1}{Z}\exp(-r(x)) = \frac{1}{Z}\exp(-\sum_{c\in S}\varphi_c(x)) \tag{11}$$

$\varphi_c(x)$ is a potential function that depends solely on the pixel values located within the clique c, with S representing the set of all cliques. By defining $\varphi_c(x)$ as a function of the image's derivative and incorporating a Gaussian noise prior, we establish a smooth prior for MRF recovery termed TM. However, MRF can exhibit greater flexibility in capturing the local properties of images.

Analytical Continuation of CSR. We present the proof of the analytical continuation of CSR. Assume that the object, such as the fluorescence distribution, has a finite spatial extent, meaning it vanishes outside a known finite region. This property can be incorporated into the imaging equation through a profile function:

$$y(r) = \int psf(r,r')x(r')J(r')\mathrm{d}r' + e \tag{12}$$

Here $J(r)$ is the profile function that defines the spatial extent of the object. For instance, $J(r)$ can be 1 in $(-1,1)$ and 0 elsewhere, meaning $x(r')$ exists only within $(-1,1)$.

According to the Paley-Wiener theorem[14], if x is spatially limited, its spectrum $\bar{x}$ is an *entire function*, i.e., it is *analytic* for all ω. Suppose there exists another spatially limited function $x^{\#}$ with the same profile function, whose spectrum $\bar{x}^{\#}$ is also an entire function, If both functions undergo the same imaging process and produce the same observed data y, we have:

$$OTF(\omega)\cdot\left[\bar{x}(\omega) - \bar{x}^{\#}(\omega)\right] = \bar{y}(\omega) - \bar{y}(\omega) = 0$$

(13)

The difference between two entire functions is still an entire function. Hence $\bar{x} - \bar{x}^{\#}$ is an entire function and equals zero within $[-\omega_l,\omega_l]$. By the Paley-Wiener theorem, if an entire function is zero over any interval, it is identically zero everywhere. This implies $\bar{x} - \bar{x}^{\#} = 0$ throughout the whole frequency domain, proving that there cannot exist another spatially limited function $x^{\#}$ that produces the same observed data. Therefore, the out-of-band extrapolation is unique. In mathematical terms, the *analytic continuation* of $\bar{x}$ outside $[-\omega_l,\omega_l]$ is unique, provided that it exists.

If the profile function is Gaussian or decreases sufficiently fast at infinity, uniqueness still holds. However, for band-limited profile functions, this conclusion does not apply[15]. Alternatively, spatial limitations can also be defined by illumination, such as a Gaussian-profile laser spot.

The Analytical Continuation of CSR shows that, in principle, the spectrum can be infinitely extrapolated. However, this derivation neglects the critical factor of noise. Noise is not necessarily an entire function, and it is always possible to find an entire function that is arbitrarily small within $[-\omega_l,\omega_l]$ but arbitrarily large outside this range. his implies that small errors in the data spectrum can lead to vastly different analytic continuations. Consequently, infinite out-of-band extrapolation is infeasible without exact data.

The Mathematical Meaning of PSWFs. Another practical and equivalent interpretation of Analytic Continuation is through the singular system representation of linear systems. Imaging can be expressed as a linear system

$$y = Lx + e \tag{14}$$

where L is the *imaging operator* which maps the true image on its noise-free data defined by imaging equation. Define the adjoint operator L^* as

$$(L^* y) = \int psf^*(r,r')y(r')\mathrm{d}r' \tag{15}$$

psf^* is the complex conjugate of psf. The singular system of operator L is the set of the triples$\{\sigma_n,u_n,v_n\}$ $n = 0,1......$ as

$$Lv_n = \sigma_n u_n;\ L^* u_n = \sigma_n v_n \tag{16}$$

With order $\sigma_0 \geq \sigma_1 \geq \sigma_2$......The singular functions or vectors $\{u_n\}$, is eigenvectors of LL*, constitute an orthonormal basis in the space of all possible noise-free data. But singular functions or vectors $\{v_n\}$, eigenvectors of L^*L, form an orthonormal basis in the set of all true images if and only if the equation $\mathrm{Lf} = 0$ has only the trivial solution$\mathrm{f} = 0$.

The following representations hold true:

$$\begin{aligned} L^* y &= \sum_{n=0}^{\infty} \sigma_n \langle y, u_n \rangle v_n \\ Lx &= \sum_{n=0}^{\infty} \sigma_n \langle x, v_n \rangle u_n \end{aligned} \tag{17}$$

If the spatial extent is finite (e.g. in $[-1,1]$) and the OTF is a rectangular function, under circle aperture, the imaging operator simplifies to:

$$(Lx)(r) = \int_{-1}^{1} \frac{\sin \omega_l (r - r')}{\pi (r - r')} \mathrm{d}r' \tag{18}$$

This is a special case of imaging process. It is proven that this integral operator is a compact operator from the space $L^2(-R,R)$ of square-integrable functions on $[-\mathrm{R},\mathrm{R}]$ into the space L^2 $(-\infty,\infty)$ of square-integrable functions on the real line[16]. Moreover, if the inverse operator exists, it effectively reaffirms the uniqueness of out-of-band extrapolation, similar to the concept of Analytic Continuation.

For this linear system (18), in 1961, Slepian and Pollack demonstrated[17]:

$$u_n(r) = \psi_n(c,r);\ v_n(r) = \frac{1}{\sigma_n} \psi_n(c,r) \tag{19}$$

Here $\psi_n(\mathrm{c,r})$ is *Prolate spheroidal wave functions* (PSWF) or *Slepin functions.* where c is the space–bandwidth parameter determined by ω_l.This is one of special functions.

Gerchberg–Papoulis Algorithm (GP). Suppose the finite spatial extent is the interval $[-1,1]$ and the band-limit is the interval $[-\omega_l,\omega_l]$:

1. GP start from the noisy data $\bar{\mathrm{y}}(\omega)$, of which the spectrum in $[-\omega_l,\omega_l]$ is noisy version of true signal and the spectrum out that interval is 0.
2. The initial model signal estimate $x^{*(1)}(r)$ is obtained by truncating $\mathrm{y}(\mathrm{r})$ to the spatial extent $[-1,1]$.
3. Then the Fourier transform $\bar{x}^{*(1)}(\omega)$ is computed and the values of this function in the interval $[-\omega_l,\omega_l]$ are replaced by the known values of $\bar{\mathrm{y}}(\omega)$.
4. The inverse Fourier transform of the resulting composite function, truncated to the interval $[-1,1]$, forms the new model signal estimate $x^{*(2)}(r)$,
5. and so on.

If we introduce the indicator function:

$$\mathbf{1}(i) = \begin{cases} 1, & |i| \leq 1 \\ 0, & |i| > 1 \end{cases} \tag{20}$$

this iterative procedure can be written as following:

$$\begin{cases} x^{*(1)}(r) = \mathbf{1}(r) y(r) \\ \overline{y}^{(k+1)}(\omega) = \overline{y}(\omega) + \left[1 - \mathbf{1}(\frac{\omega}{\omega_l}) \right] x^{*(k)}(\omega) \\ x^{*(k+1)} = \mathbf{1}(r) y^{(k+1)}(r) \end{cases} \tag{21}$$

GP is a special case of *projections onto convex sets* (POCS)[18], an alternating projection method to find a point in the intersection of two closed convex sets. In the absence of noise, GP theoretically converges to the correct solution, as indicated by the uniqueness implied by analytic continuation[19]. However, in the presence of noise, the algorithm risks overfitting. Early termination often yields better results, while adding regularization can further suppress noise especially based on POCS framework[20].

For a more detailed mathematical description of the above three sections, refer to extensive bibliography provided therein[20].

Richardson-Lucy Deconvolution Detail. What many may not know is that RL and the standard reconstruction algorithm in Positron Emission Tomography (PET), Maximum Likelihood Expectation Maximization (MLEM)[21], are

essentially the same algorithm, despite MLEM being proposed years later. The name MLEM clearly illustrates the characteristics of this algorithm: it assumes the noise in the data y follows a Poisson distribution, models the optimization objective based on Maximum Likelihood, and solves it using Expectation-Maximization (EM) optimization. Interested readers can refer to Ref. [22] for a detailed derivation.

However, there is another way to interpret RL, which shows that assuming a Poisson noise prior is not necessary, making the connection with CSR clearer. Define the generalized *Kullback-Leibler Divergence* between two n-dimensional vectors, $\mathbf{y}$ and $\mathbf{c}$, as:

$$\mathrm{KL}(\mathrm{y};\mathrm{c}) = \sum_{i=1}^{n} \mathrm{y}_i \ln \frac{\mathrm{y}_i}{\mathrm{c}_i} - \mathrm{y}_i + \mathrm{c}_i \tag{22}$$

This divergence acts as a distance measure. If we replace the ℓ_2 norm in the Least Squares (LS) problem (associated with Landweber iteration) with generalized Kullback-Leibler Divergence, we obtain a special case of the basic framework where only the data fidelity term is considered, without including a regularization term:

$$\mathrm{x}^* = \arg\min_{\mathrm{z}} \mathrm{KL}(\mathrm{y}; \mathbf{P}\cdot \mathrm{z}) \tag{23}$$

Thus, RL can therefore be viewed as an iterative algorithm for solving this optimization objective—a specific form of gradient descent. It has been proven that RL converges to the minimizer[23]. This refutes the misunderstanding that RL cannot guarantee fidelity[24]. Moreover, in this interpretation, no Poisson noise assumption is necessary. In fact, Richardson did not assume Poisson noise in his original paper but instead derived RL from a Bayesian model.

Mathematical Concepts in Sparsity Prior. The following discussion is primarily based on the excellent books[25].

ℓ_0 minimization. For ℓ_0 minimization, suppose $||\mathbf{x}||_0 \le k$, as long as the matrix $\mathbf{A}$ satisfies the following property:

$$\text{the only } \boldsymbol{\delta} \in \mathrm{null}(\mathbf{A}) \text{ with } \|\boldsymbol{\delta}\|_0 \le 2k \text{ is } \boldsymbol{\delta} = \mathbf{0}$$

Then the solution to ℓ_0 minimization is unique and corresponds to the true solution. Since null space properties are difficult to measure directly, an equivalent formulation is as follows: we define the *Kruskal rank* of a matrix $\mathbf{A}$, denoted as $\mathrm{Krank}(\mathbf{A})$, as the largest integer r, such that every subset of r columns of $\mathbf{A}$ is linearly independent. If

$$\|\mathrm{x}\|_0 \le \frac{1}{2}\mathrm{Krank}(\mathbf{A}) \tag{24}$$

then the conclusion remains the same: the solution to ℓ_0 minimization is unique and corresponds to the true signal. For detailed proofs, refer to Ref.[25a] chapter 2.

ℓ_1 minimization. For a matrix $\mathbf{A}$ with $\boldsymbol{a}_i$ as its nonzero column. We define *Mutual Coherence* as the largest normalized inner product between two distinct columns**:**

$$\mu(\mathbf{A}) = \max_{i\neq j} \left| \left\langle \frac{a_i}{\|a_i\|_2}, \frac{a_j}{\|a_j\|_2} \right\rangle \right| \tag{25}$$

The mutual coherence is directly related to Kruskal rank:

$$\mathrm{Krank}(\mathbf{A}) \ge \frac{1}{\mu(\mathbf{A})} \tag{26}$$

Thus, provided $\mu(\mathbf{A})$ is small enough, ℓ_0 minimization will uniquely recover true siagnl.

Treat mutual coherence as bridge to connect ℓ_0 minimization and ℓ_1 minimization. As if:

$$\|\mathrm{x}\|_0 \le \frac{1}{2\mu(\mathbf{A})} \tag{27}$$

Then, true signal $\mathbf{x}$ is the unique solution of ℓ_1 minimization. All the detailed proofs are given in Ref.[25a] chapter 3.

Restricted Isometry Property. Define s-sparse vectors:Σ_s:= $\{\mathbf{x} \in \mathbb{R}^n | \mathbf{x} \text{ has at most } s \text{ non-zero elements}\}$. The sth Restricted Isometry Constant (RIC) δ_s of a matrix $\mathbf{A} \in \mathbb{R}^{m\times n}$ is the smallest $\delta \ge 0$ such that

$$(1-\delta)\|\mathrm{x}\|_2^2 \le \|\mathbf{A}\cdot \mathrm{x}\|_2^2 \le (1+\delta)\|\mathrm{x}\|_2^2 \quad \forall\, \mathrm{x} \in \Sigma_s \tag{28}$$

If $0 < \delta_s < 1$ then the matrix $\mathbf{A}$ is said to have the Restricted Isometry Property (RIP) of order s.

RIP implies accurate and stable recovery for QCBP: Suppose true signal $\mathbf{x}$ is compressible and can be approximate best by exact$s$-sparse signal $[x]_s$, and y=$\mathbf{A} \cdot \mathbf{x} + \mathbf{e}$, with $\mathbf{A} \in \mathbb{R}^{m \times n}$ and $\eta(e)$ is noise level. If $\mathbf{A}$ has the RIP of order $2s$ with constant $\delta_{2s} < \sqrt{2} - 1$, then any minimizer $\mathbf{x}^*$ of QCBP and true signal $\mathbf{x}$ have:

$$\left\|x^* - x\right\|_2 \leq C \frac{\left\|x - [x]_s\right\|_1}{\sqrt{s}} + C'\eta(e) \tag{29}$$

For constant C and C' that depend only with δ_{2s}

RIP Matrix. If Matrix $\mathbf{A} \in \mathbb{R}^{m \times n}$ is randomly chosen rows of $n \times n$ Fourier Matrix——Random means that there is a probability that each row of the Fourier Matrix be selected——If:

$$m \geq \frac{C}{\delta^2} s \log^4(n) \tag{30}$$

then with high probability, $\mathbf{A}$ satisfies the RIP of order s, with constant $\delta_s < \delta$. Combine previous theory, in this point, QCBP could accurate and stable recovery compressible true signal.

A randomly chosen Fourier matrix is a special case of more general random matrices, such as those composed of randomly chosen rows of a unitary matrix. The conclusion remains the same with an adjusted constant.

This concept is crucial for sparse coding with wavelets because the DWT matrix $\mathbf{\Psi}$ is also a unitary matrix. Since the product of two unitary matrices is also unitary, we can transform a signal into the wavelet domain to apply CS theory. The sensing matrix becomes $\mathbf{A\Psi}$**.**

In addition to randomly chosen rows of a unitary matrix, two other types of random matrices have been found to satisfy the RIP under probabilistic conditions: random Gaussian matrices and random Bernoulli matrices. The minimum number of samples required varies depending on the specific type of random matrix used.

Sparse Points SR. In a 1992 work, Donoho introduced a sparsity model different from ℓ_0 norm sparsity, where an image is represented by a set of sparse points[26]. He considered the problem of resolving two points on a one-dimensional grid and demonstrated that if the true solution satisfies specific sparsity constraints (related to point spacing), the solution x^* obtained through ℓ_1 minimization has an error relative to the true signal x that depends on the sparsity, super-resolution factor, and noise level. If this error is sufficiently small, x^* and x are nearly identical, implying that super-resolution is achievable.

However, Donoho assumed the existence of such an algorithm and analyzed an exhaustive search algorithm, which is computationally infeasible. The question of whether a practical and stable algorithm for sparsity-prior CSR exists was addressed later by Morgenshtern and Candes in 2016[27]. They extended Donoho's results to higher dimensions and proposed a feasible linear programming algorithm.

The latest SR theory models objects as a collection of Dirac delta functions, where super-resolution pertains to precise localization. This concept has a long history in signal processing, tracing back to the 19th century, where signals are treated as weighted sums of sinusoidal components. SR aims to distinguish closely spaced sinusoids. For more details, readers may refer to standard textbooks[28] and the paper [29].

Generalized Sparsity prior. A final practical consideration is how to apply CS when a signal is neither sparse nor compressible in its raw form. In such cases, CS can still be applied by identifying a suitable transformation T that converts the raw signal into a domain where it exhibits sparsity. This approach, known as a *sparse transform*, can be expressed as $b = \mathbf{T}\mathbf{x}$, where $||b||_1$ is small. Sparse transforms establish a connection between TV, DWT, and sparsity priors.

The success of CS, particularly the application of the ℓ_1 norm, has inspired CSR methods that

leverage models incorporating generalized form of sparsity, or *sparsity regularization*.

Analysis Regularization. The first type of sparse regularization relies on a sparse transform $\mathbf{T}$ to exploit the sparsity of the true signal, with the framework given as:

$$x^* = \arg\min_z \|\mathbf{P}\cdot z - y\|_2^2 + \lambda\|\mathbf{T}\cdot z\|_1 \tag{31}$$

This is called *analysis regularization*. Classical examples include TV and DWT. Analysis regularization has also spurred the exploration of general low-dimensional models[30].

Sparse Coding. In contrast to signal transforms, another form of sparsity assumes that the true signal is derived from another domain. The core idea is to represent the true signal x as a linear combination of a few elements (referred to as "*atoms*" in the literature) from a dictionary $\mathbf{D}$:$x \approx Dz$, where z is the coefficient vector that is encouraged to be sparse. The symbol $\approx$ indicates an approximation related to compressibility. This approach is referred to as the *sparse representation* of x, and the process of determining a suitable dictionary $\mathbf{D}$ for achieving this sparse representation is known as the *sparse coding problem*.

Given set $\mathbf{D}$ as prior**,** the sparsity regularization is applied to coefficients:

$$\begin{aligned} x^* &= \mathbf{D}\cdot z^* \\ z^* &= \arg\min_z \frac{1}{2}\|\mathbf{P}\cdot\mathbf{D}\cdot z\|_2^2 + \lambda\|z\|_1 \end{aligned} \tag{32}$$

This is called *synthesis regularization.* Here, the combination is exact, which may present flaws in practice. An alternative synthesis formulation that allows for an approximate sparsity model is:

$$\begin{cases} x^* = \arg\min_{z^*} \frac{1}{2}\|\mathbf{P}\cdot z^*\|_2^2 + \beta R(z^*) \\ R(z^*) = \min_z \frac{1}{2}\|z^* - \mathbf{D}\cdot z\|_2^2 + \alpha\|z\|_1 \end{cases} \tag{33}$$

When $\mathbf{D}$ and $\mathbf{T}$ are both square and full-rank, analysis and synthesis is the same as $\mathbf{D} = \mathbf{T}^{-1}$. However, usually, $\mathbf{T}$ is a general operator and $\mathbf{D}$ is overcomplete. Often, $\mathbf{D}$ represents a large set of basis functions and is commonly referred to as an (overcomplete) *dictionary.*

Dictionary Learning. One significant challenge in sparse coding is that images are inherently high-dimensional vectors, making the decomposition of such high-dimensional data extremely difficult. Finding an appropriate dictionary purely through mathematical methods is a considerable challenge.

Research on image statistics suggests that image patches can be effectively represented as sparse linear combinations of elements from a carefully constructed set of real image patches. This sparse representation is intended to work for both low-resolution and high-resolution images.

A milestone in this direction is the work titled "Image Super-Resolution Via Sparse Representation" (2010)[31]. Based on CS theory, this study jointly trains two dictionaries: one for low-resolution image patches and another for high-resolution patches. The sparse representations of corresponding low- and high-resolution patches with respect to their own dictionaries are constrained to be similar. This ensures that the sparse representation of a low-resolution patch can be applied to the high-resolution dictionary to generate a high-resolution patch. This approach has demonstrated effectiveness in both general image SR and specialized tasks such as face hallucination.

In fact, this work represents one of the earliest examples of using data to automatically learn priors, a framework known as dictionary learning[32]. The training of patch sets in this context is similar to Deep Learning.

In microscopy, dictionary learning is applied in Light field microscopy (LFM), which suffers from artifact contamination due to the ill-posed nature of the reconstruction problem inherent in RL deconvolution[33]. Dictionary LFM improves noise robustness by using an overcomplete dictionary trained from real data.

Operator Splitting Optimization Methods. The emergence of non-linear regularization has introduced a unique class of optimization problems.

Taking ℓ_1 minimization as an example, the QCBP can be reformulated into the well-known LASSO (Least Absolute Shrinkage and Selection Operator) problem using the Lagrange multiplier method:

$$x^* = \arg\min_{z} \|\mathbf{A} \cdot z - y\|_2^2 + \lambda \|z\|_1 \quad (34)$$

Many non-linear regularizations, such as TV, share a similar structure: the regularization term is convex but non-smooth (e.g., the ℓ_1 norm in LASSO). This non-smoothness renders traditional gradient-based methods unsuitable. Readers familiar with optimization theory might consider subgradient methods as an alternative. However, modern image processing tasks often involve large-scale problems—for instance, typical microscopic images can be 1024x1024 pixels or larger. Traditional convex optimization methods, such as subgradient descent, are extremely slow, with a convergence rate of $O(\frac{1}{\sqrt{k}})$. To make matters worse, linear operators in the regularization term (e.g., TV) can make computing the subgradient itself challenging.

Around the 2010s, the demand for efficient LASSO optimization led to the development of a new class of algorithms known as *operator splitting optimization methods*. These algorithms leverage a specialized mathematical tool called the *proximal operator*. For a convex function f (under certain additional conditions, which are omitted here), the proximal operator at any vector x with parameter α is defined as the solution to the following optimization problem:

$$\text{prox}_{\alpha f}(x) := \arg\min_{u} \left\{ f(u) + \frac{1}{2\alpha} \|u - x\|_2^2 \right\} \quad (35)$$

This optimization problem has a unique solution. For the ℓ_1 norm, the proximal operator is known as the *shrinkage operator*:

$$\text{shrink}_{\alpha}(x)_i = \left(|x_i| - \alpha\right)_+ \text{sgn}(x_i) \quad (36)$$

The key insight is that for many non-linear regularizations, the proximal operator is computationally efficient to evaluate. The operator splitting method employs a divide-and-conquer strategy: for the smooth data fidelity term, its gradient is computed, while for the non-smooth regularization term, its proximal operator is computed. These steps are alternated in an iterative minimization process, thus avoiding the need to address both smooth and non-smooth terms simultaneously. These algorithms are guaranteed to converge and are relatively fast, making them the standard approach for solving non-linear regularization optimization problems.

Among various operator splitting algorithms, one of the most notable is ADMM[34] (Alternating Direction Method of Multipliers). Initially when applied in ℓ_1 minimization referred to as the *alternating split Bregman algorithm*, it was derived from the perspective of Bregman distance[35]. Later, it was recognized as a rediscovery of the ADMM method, originally proposed decades earlier. Applying ADMM to ℓ_1 minimization yields the ISTA (Iterative Shrinkage Thresholding Algorithm) and its accelerated version, FISTA (Fast Iterative Shrinkage-Thresholding Algorithm) [36]:

$$x^{(k+1)} = \text{shrink}_{\lambda t}(x^{(k)} - 2t\mathbf{A}^{\mathrm{T}}(\mathbf{A} \cdot x^{(k)} - y)) \quad (37)$$

The introduction of FISTA significantly advanced CS theory, which in turn further stimulated the development of operator splitting methods. For more detailed mathematical discussions on operator splitting methods, readers may refer to the review[37].

If the regularization term is a non-convex function (e.g., ℓ_0 minimization), there are generally no universal solution methods. Custom optimization strategies are often required for specific cases, which can be very challenging and remain an active area of research[38].

Although optimization theory can be complex, many mature software tools have simplified these algorithms into "plug-and-play" solutions, such as DeconvolutionLab2[39] and GlobalBioIm[40].

Parameters Tuning. Another critical challenge in optimization is parameter tuning, such as the λ

in the basic function, which determines the relative importance of each term. Since the advent of inverse problem approach, this has remained an unsolved issue. In practice, parameters are often determined by experimental testing. Although there are many theories about automatic parameter tuning, they are beyond the scope of this review[41].

1. Sung Cheol, P.; Min Kyu, P.; Moon Gi, K., Super-resolution image reconstruction: a technical overview. *IEEE Signal Processing Magazine* **2003,** *20* (3), 21-36.

2. Maiseli, B.; Abdalla, A. T., Seven decades of image super-resolution: achievements, challenges, and opportunities. *EURASIP Journal on Advances in Signal Processing* **2024,** *2024* (1), 78.

3. Tsai, R. Y.; Huang, T. S., Multiframe image restoration and registration. *Multiframe image restoration and registration* **1984,** *1*, 317-339.

4. Milanfar, P., *Super-resolution imaging*. CRC press: 2017.

5. Chen, H.; He, X.; Qing, L.; Wu, Y.; Ren, C.; Sheriff, R. E.; Zhu, C., Real-world single image super-resolution: A brief review. *Information Fusion* **2022,** *79*, 124-145.

6. Mertz, J., *Introduction to Optical Microscopy*. 2 ed.; Cambridge University Press: Cambridge, 2019.

7. Unser, M., Sampling-50 years after Shannon. *Proceedings of the IEEE* **2000,** *88* (4), 569-587.

8. Heintzmann, R.; Sheppard, C. J. R., The sampling limit in fluorescence microscopy. *Micron* **2007,** *38* (2), 145-149.

9. Hansen, P. C.; Nagy, J. G.; O'Leary, D. P., *Deblurring Images: Matrices, Spectra, and Filtering (Fundamentals of Algorithms 3) (Fundamentals of Algorithms)*. Society for Industrial and Applied Mathematics: 2006.

10. Boyd, S.; Vandenberghe, L., *Convex Optimization*. Cambridge University Press: Cambridge, 2004.

11. van de Schoot, R.; Depaoli, S.; King, R.; Kramer, B.; Märtens, K.; Tadesse, M. G.; Vannucci, M.; Gelman, A.; Veen, D.; Willemsen, J.; Yau, C., Bayesian statistics

and modelling. *Nature Reviews Methods Primers* **2021,** *1* (1), 1.

12. Geman, S.; Geman, D., Stochastic Relaxation, Gibbs Distributions, and the Bayesian Restoration of Images. *IEEE Transactions on Pattern Analysis and Machine Intelligence* **1984,** *PAMI-6* (6), 721-741.

13. Stuart, A. M., Inverse problems: A Bayesian perspective. *Acta Numerica* **2010,** *19*, 451-559.

14. Helly, Fourier transforms in the complex domain. *Monatshefte für Mathematik und Physik* **1936,** *44* (1), A8-A9.

15. Bertero, M.; Brianzi, P.; Pike, E. R., Super-resolution in confocal scanning microscopy. *Inverse Problems* **1987,** *3* (2), 195.

16. Bertero, M.; Mol, C. D.; Pike, E. R., Linear inverse problems with discrete data: II. Stability and regularisation. *Inverse Problems* **1988,** *4* (3), 573.

17. Slepian, D. S.; Pollak, H. O., Prolate spheroidal wave functions, fourier analysis and uncertainty — II. *Bell System Technical Journal* **1961,** *40*, 43-63.

18. Youla, D. C.; Webb, H., Image Restoration by the Method of Convex Projections: Part 1[illegible]Theory. *IEEE Transactions on Medical Imaging* **1982,** *1* (2), 81-94.

19. De Santis, P.; Gori, F., On an Iterative Method for Super-resolution. *Optica Acta: International Journal of Optics* **1975,** *22* (8), 691-695.

20. Lindberg, J., Mathematical concepts of optical superresolution. *Journal of Optics* **2012,** *14* (8), 083001.

21. Shepp, L. A.; Vardi, Y., Maximum Likelihood Reconstruction for Emission Tomography. *IEEE Transactions on Medical Imaging* **1982,** *1* (2), 113-122.

22. Chapter 17: Poisson Data Models. In *Foundations of Computational Imaging: A Model-Based Approach*, pp 287-303.

23. Salvo, K.; Defrise, M., A Convergence Proof of MLEM and MLEM-3 With Fixed Background. *IEEE Transactions on Medical Imaging* **2019,** *38* (3), 721-729.

24. Hou, Y. W.; Wang, W. Y.; Fu, Y. Z.; Ge, X. C.; Li, M. Q.; Xi, P., Multi-resolution analysis enables fidelity-ensured deconvolution for fluorescence microscopy. *Elight* **2024,** *4* (1).

25. (a) Wright, J.; Ma, Y., *High-Dimensional Data Analysis with Low-Dimensional Models: Principles, Computation, and Applications*. Cambridge University Press: Cambridge, 2022; (b) Adcock, B.; Hansen, A. C., *Compressive Imaging: Structure, Sampling, Learning*. Cambridge University Press: Cambridge, 2021.

26. Donoho, D. L., SUPERRESOLUTION VIA SPARSITY CONSTRAINTS. *Siam Journal on Mathematical Analysis* **1992,** *23* (5), 1309-1331.

27. Morgenshtern, V. I.; Candès, E. J., Super-Resolution of Positive Sources: The Discrete Setup. *SIAM Journal on Imaging Sciences* **2016,** *9* (1), 412-444.

28. Stoica, P., *Spectral analysis of signals*. 2004; Vol. 452.

29. Candès, E. J.; Fernandez-Granda, C., Towards a Mathematical Theory of Super-resolution. *Communications on Pure and Applied Mathematics* **2014,** *67* (6), 906-956.

30. Carin, L.; Baraniuk, R. G.; Cevher, V.; Dunson, D.; Jordan, M. I.; Sapiro, G.; Wakin, M. B., Learning Low-Dimensional Signal Models. *IEEE Signal Processing Magazine* **2011,** *28* (2), 39-51.

31. Yang, J.; Wright, J.; Huang, T. S.; Ma, Y., Image Super-Resolution Via Sparse Representation. *IEEE Transactions on Image Processing* **2010,** *19* (11), 2861-2873.

32. Tošić, I.; Frossard, P., Dictionary Learning. *IEEE Signal Processing Magazine* **2011,** *28* (2), 27-38.

33. Zhang, Y.; Xiong, B.; Zhang, Y.; Lu, Z.; Wu, J.; Dai, Q., DiLFM: an artifact-suppressed and noise-robust light-field microscopy through dictionary learning. *Light: Science & Applications* **2021,** *10* (1), 152.

34. Stephen, B.; Neal, P.; Eric, C.; Borja, P.; Jonathan, E., *Distributed Optimization and Statistical Learning via the Alternating Direction Method of Multipliers*. now: 2011; p 1.

35. Benning, M.; Riis, E. S., Bregman Methods for Large-Scale Optimization with Applications in Imaging. In *Handbook of Mathematical Models and Algorithms in Computer Vision and Imaging: Mathematical Imaging and Vision*, Chen, K.; Schönlieb, C.-B.; Tai, X.-C.; Younces, L., Eds. Springer International Publishing: Cham, 2021; pp 1-42.

36. Beck, A.; Teboulle, M., A Fast Iterative Shrinkage-Thresholding Algorithm for Linear Inverse Problems. *Siam J Imaging Sciences* **2009,** *2* (1), 183-202.

37. (a) Chambolle, A.; Pock, T., An introduction to continuous optimization for imaging. *Acta Numerica* **2016,** *25*, 161-319; (b) Condat, L.; Kitahara, D.; Contreras, A.; Hirabayashi, A., Proximal Splitting Algorithms for Convex Optimization: A Tour of Recent Advances, with New Twists. *SIAM Review* **2023,** *65* (2), 375-435.

38. Sui, X.; He, Z.; Chu, D.; Cao, L., Non-convex optimization for inverse problem solving in computer-generated holography. *Light: Science & Applications* **2024,** *13* (1), 158.

39. Sage, D.; Donati, L.; Soulez, F.; Fortun, D.; Schmit, G.; Seitz, A.; Guiet, R.; Vonesch, C.; Unser, M., DeconvolutionLab2: An open-

source software for deconvolution microscopy. *Methods* **2017,** *115*, 28-41.

40. Soubies, E.; Soulez, F.; McCann, M. T.; Pham, T.-a.; Donati, L.; Debarre, T.; Sage, D.; Unser, M., Pocket guide to solve inverse problems with GlobalBioIm. *Inverse Problems* **2019,** *35* (10), 104006.

41. (a) Stone, M., Cross-validation:a review 2. *Series Statistics* **1978,** *9* (1), 127-139; (b) Golub, G. H.; Heath, M.; Wahba, G., Generalized Cross-Validation as a Method for Choosing a Good Ridge Parameter. *Technometrics* **1979,** *21* (2), 215-223; (c) Phillips, D. L., A Technique for the Numerical Solution of Certain Integral Equations of the First Kind. *J. ACM* **1962,** *9* (1), 84–97; (d) Saquib, S. S.; Bouman, C. A.; Sauer, K., ML parameter estimation for Markov random fields with applications to Bayesian tomography. *IEEE Transactions on Image Processing* **1998,** *7* (7), 1029-1044.
